# BiFeO_3_-Based Relaxor Ferroelectrics for Energy Storage: Progress and Prospects

**DOI:** 10.3390/ma14237188

**Published:** 2021-11-25

**Authors:** Bipul Deka, Kyung-Hoon Cho

**Affiliations:** 1 Research Institute of Advanced Materials, Kumoh National Institute of Technology, Gumi 39177, Korea; 2 School of Materials Science and Engineering, Kumoh National Institute of Technology, Gumi 39177, Korea; 3 Department of Physics, Pub Kamrup College, Kamrup, Assam 781381, India

**Keywords:** energy storage, BiFeO_3_, relaxor ferroelectrics, domain engineering, polymorphic nanodomain

## Abstract

Dielectric capacitors have been widely studied because their electrostatic storage capacity is enormous, and they can deliver the stored energy in a very short time. Relaxor ferroelectrics-based dielectric capacitors have gained tremendous importance for the efficient storage of electrical energy. Relaxor ferroelectrics possess low dielectric loss, low remanent polarization, high saturation polarization, and high breakdown strength, which are the main parameters for energy storage. This article focuses on a timely review of the energy storage performance of BiFeO_3_-based relaxor ferroelectrics in bulk ceramics, multilayers, and thin film forms. The article begins with a general introduction to various energy storage systems and the need for dielectric capacitors as energy storage devices. This is followed by a brief discussion on the mechanism of energy storage in capacitors, ferroelectrics, anti-ferroelectrics, and relaxor ferroelectrics as potential candidates for energy storage. The remainder of this article is devoted to reviewing the energy storage performance of bulk ceramics, multilayers, and thin films of BiFeO_3_-based relaxor ferroelectrics, along with a discussion of strategies to address some of the issues associated with their application as energy storage systems.

## 1. Introduction

Global warming poses potential threats to the planet Earth’s future. The continuous burning of fossil fuels has increased the concentration of CO_2_ and other greenhouse gases in the Earth’s atmosphere, leading to a warmer atmosphere and climate change. In addition, the depletion of fossil fuel resources, the dominant candidate in the energy market, poses the risk of an energy crisis in a world where the number of consumers is increasing day by day. Considering the serious threats to the lives on Earth and the risk of an energy crisis posed by the use of fossil fuel resources, the transition to clean energy is a serious consideration. However, renewable energy sources such as solar, wind, tides, and geothermal energy are intermittent by nature. Therefore, harnessing and storing renewable energy for future access is a challenging task.

Electrical energy harvested from renewable sources offers enormous opportunities for meeting future energy demands and the feasibility of the transition to clean energy. However, the usefulness of the electrical energy generated depends on its efficient storage, which is necessary for around-the-clock use. An efficient electrical energy storage (EES) system is the heart of the commercial and residential grid-based utilization of electrical energy. Therefore, the development of advanced EES systems is critically important for meeting the growing energy demands and effectively leveling the cyclic nature of such energy sources [1]. For over 200 years, batteries have been widely used in EES systems and are still being widely used. Solid oxide fuel cells (SOFCs), electrochemical capacitors (ECs), superconducting magnetic energy storage (SMES) systems, flywheels, and electrostatic capacitors (dielectric capacitors) are common current energy storage technologies [2].

**Figure 1:**
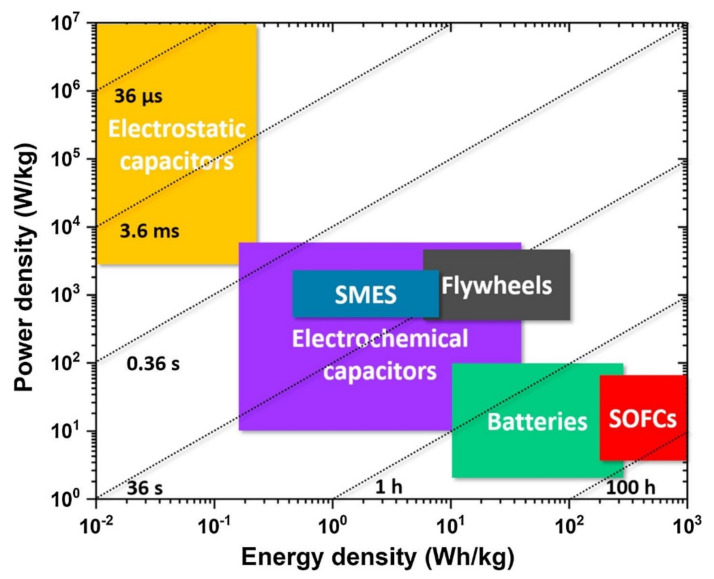
Ragone plot of various energy storage devices: electrostatic capacitors, electrochemical capacitors, SMES, flywheels, batteries, and SOFCs. The straight dashed lines and the associated times correspond to the characteristic times. Reused with permission from [2]. © 2021 Elsevier Ltd.

A perfect energy storage device is characterized by high energy and power densities. A comparison of the storage efficiency of the technologically relevant candidates for EES systems can be realized from the Ragone plot shown in Figure 1, which displays the status of EES systems according to their energy and power densities. As can be seen, SOFCs and batteries exhibit a high energy density with low power density, while dielectric capacitors exhibit the opposite behavior, that is, high power density and low energy density. The ECs, SMES, and flywheel have medium power and energy densities. In addition to the high power and energy density, the charge/discharge rate is a deciding factor for EES systems. The energy storage and delivery in SOFCs/batteries are based on the chemical reaction process and may take 1–100 h of time. Dielectric capacitors typically exhibit fast charge/discharge rates, between μs and ms, whereas those for ECs, flywheels, and SMES are between 1 s and 1 h. The fast charge/discharge rate of dielectric capacitors is associated with the separation of comparatively fewer heavy bound charges under the influence of the electric field to be stored. Thus, to summarize, no individual EES candidate possesses both high power and energy densities simultaneously. Therefore, the technological relevance of each candidate as an EES may be determined by the final requirements. However, among the various potential candidates for EES, dielectric capacitors have the advantage of withstanding high-voltage and large-scale applications because of their lower cost [3,4].

Dielectric capacitors are one of the key components in modern communication technology, with applications in electronic circuits, warfare, distributed power systems, hybrid electric vehicles, clean energy storage, high-power applications, etc. [5,6,7,8,9,10,11]. Among the various EES systems, dielectric capacitors exhibit the fastest discharge speed; therefore, they can generate intense pulse power [11,12,13,14]. The fast discharge speed and high fatigue resistance of dielectric capacitors enable their potential applications in various electronic systems. This includes medical equipment such as defibrillators, pacemakers, surgical lasers, and X-ray units; scientific research equipment such as high-power accelerators and high-intensity magnetic fields; commercial system devices such as camera flash, underground oil and gas exploration, avionics, transportation (hybrid cars, space shuttle power systems), transversely excited atmospheric lasers, and advanced electromagnetic systems [15].

The current review discusses the recent progress on the development of high-energy storage dielectric capacitors based on the relaxor ferroelectric (RFE) of BiFeO_3_. The two important figures of a capacitor that determine its energy storage performance are the recoverable energy density (*U*_rec_) and energy efficiency (*η*), which depend on the saturation polarization (*P*_max_), remnant polarization (*P*_r_), and breakdown strength (BDS) of the materials. Linear dielectric (LD), ferroelectric (FE), and anti-ferroelectric (AFE) materials are widely used for the fabrication of ceramic capacitors. Although the LDs possess excellent values of BDS, their *U*_rec_ values are quite low due to weak polarization. FE materials, on the other hand, possess quite a large *P*_max_ and *P*_r_. As can be seen in Section 2, the large values of both *P*_max_ and *P*_r_ result in a low *U*_rec_ value. Moreover, the ferroelectric materials suffer large hysteresis loss, which has a significant detrimental effect on the energy efficiency of the capacitors. AFE materials behave like LDs in the low field regime, undergoing a field-induced FE state, yielding a high *P*_max_ at a high electric field with large hysteresis loss. Therefore, the issues associated with the AFE for their applications in the high-energy storage application are essentially similar to that of LDs at low electric fields and to that of FEs at high electric fields. However, the problem of hysteresis loss inherent to FE and AFE materials is found to be minimized in RFEs while maintaining a significantly large value of *P*_max_. This motivates the scientific community to turn their heads towards the RFEs in search of high-energy storage capacitors.

Ceramic dielectric capacitors based on BiFeO_3_ have recently gained interest in the field of energy storage applications because of the high polarization (~90 μC cm^−2^) predicted in BiFeO_3_, along with its high ferroelectric Curie temperature (*T*_C_) (~830 °C) [16]. The advantage of having a high *T*_C_ is that the materials do not lose their ferroelectric (FE) nature at such high temperatures, which is essential for applications in the high-temperature regime. Temperature stability is an important issue that needs to be addressed while designing a capacitor for operation in the high-temperature regime. For example, ceramic-polymer composites have excellent storage performance; however, their performance degrades very rapidly as the temperature approaches 100 °C [15]. Pb-based ferroelectrics often have the disadvantage of adverse harmful effects on the environment and human beings. Pb-oxide, which is a main component of Pb-based ferroelectrics, is highly toxic and volatile at high temperatures, causing environmental pollution during the fabrication process. Disposal and recycling of Pb-based materials and devices at an industrial scale also creates atmospheric problems due to the difficulty of Pb removal. Exposure to the heavy metal Pb causes detrimental effects such as kidney and brain damages, and chronic exposure may lead to damages to the central nervous system and affect blood pressure, vitamin D metabolism, etc. Young children are more vulnerable to Pb exposure, as the absorption of Pb in children’s bodies is 4–5 times higher than in an adult body. Therefore, to reduce the use of hazardous materials such as Pb, various countries have adopted different restrictions on hazardous materials [17,18]. Although there are a few reviews on dielectrics for energy storage in general, to the best of our knowledge, there has been no such review for BiFeO_3_-based relaxor ferroelectrics. Here, we present a review of the recent progress on BiFeO_3_-based relaxor ferroelectric for energy storage, discussing various issues to meet practical applications. We first discuss the fundamentals of energy storage in dielectrics and the pros and cons of various nonlinear dielectrics with respect to their applications in energy storage. We then discuss the characteristics of relaxor ferroelectrics and their importance in energy storage, followed by a brief discussion of the basic properties of BiFeO_3_. Following this, we present the recent progress in energy storage studies on BiFeO_3_ and strategies for further enhancement.

## 2. Fundamentals of the Energy Storage Mechanism in Dielectrics

The energy storage mechanism of dielectrics is based on their polarization under the application of an electric field. A dielectric under an applied electric field is polarized such that equal amounts of positive and negative charges accumulate at the surfaces of the dielectrics. In other words, an electric field opposite to the applied field is induced inside the dielectric. The strength of the induced field grows exponentially with time until its magnitude is equal to that of the external field. This process is known as charging the capacitor. Thus, the induced electrostatic energy is stored in the dielectric and can be used for application upon discharge through a load. The amount of stored energy (*U*) can be obtained from the potential difference (*V*) across the dielectrics and the charge (*q*) induced at the electrode on the surface of the dielectrics using the following equation:

(1) $$U=\int_{0}^{q_{max}}Vdq$$

where *q_max_* is the maximum amount of charge accumulated at the electrode when the capacitor is fully charged, and *dq* is the increment of charge during charging. A figure-of-merit (FOM), which signifies the energy storage performance of a capacitor, is represented in terms of energy storage density (*U*_st_), defined as the energy stored per unit volume. Mathematically,

(2) $$U_{st}=\frac{\int_{0}^{q_{max}}Vdq}{Ad}=\int_{0}^{D_{max}}EdD$$

where *A* is the electrode area of the capacitor, *d* is the distance between the electrodes (thickness of the dielectric layer), and *D* is the electric displacement of the capacitor. For a weak electric field, *D* is related to the external electric field (*E*) and polarization (*P*) as follows:

(3) $$D=P+\varepsilon_{0}E$$

where *ε*_0_ represents the permittivity of a vacuum. Materials obeying Equation (3) are classified as linear dielectrics. For a linear dielectric, *P* is assumed to be a linear function of *E*:

(4) $$P=\varepsilon_{0}\chi E$$

where the quantity *χ* is termed the linear dielectric susceptibility. At a high electric field, it is necessary to consider the nonlinear contribution of susceptibility, and Equation (4) takes the most general form as:

(5) $$P=\varepsilon_{0}\left(\chi E+\chi^{\left(2\right)}E^{2}+\chi^{\left(3\right)}E^{3}+\ldots \right)$$

where *χ*^(2)^ and *χ*^(3)^ are higher-order susceptibilities, giving rise to nonlinear effects. Using linear approximation, the stored energy density of a dielectric material with a high dielectric constant (*D* ≈ *P*) can be calculated as follows:

(6) $$U_{st}=\overset{P_{max}}{\underset{0}{\int }}EdP\;$$

**Figure 2:**
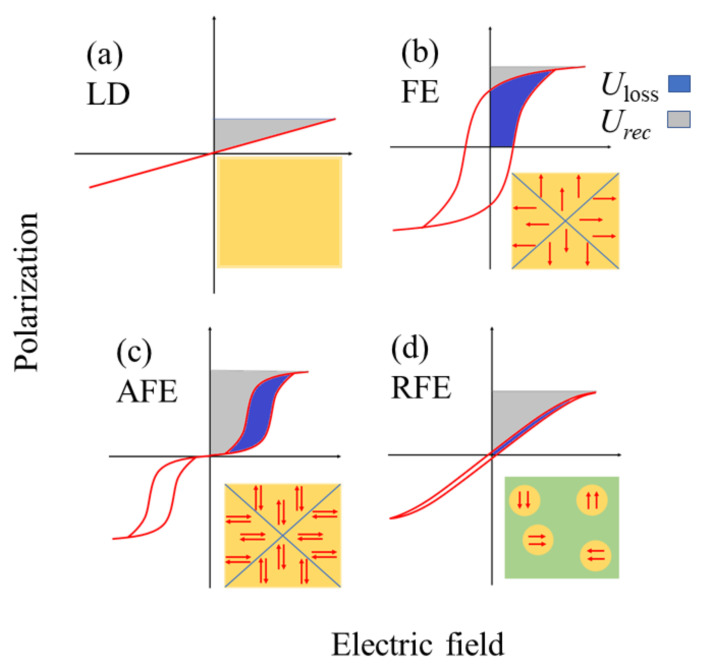
Typical *P*–*E* loops for (**a**) LD (**b**) FE, (**c**)AFE, and (**d**) RFE. Insets are schematics of domains with the alignment of polarization vectors (arrowheads). In RFEs, polar nanoregions (shown in circular patches) are sparingly distributed in a non-ferroelectric matrix (green area in the inset).

Equation (6) indicates that the electric polarization as a function of the electric field should be measured to calculate *U_st_*. In other words, it is necessary to measure the polarization-electric field (*P*−*E*) hysteresis loop to obtain the stored energy density, as shown in Figure 2. Therefore, the shape and size of the *P*−*E* loop and the nature of the dipole/domain structures determine the energy storage performance of dielectric materials. However, the dynamics of the polarization vector, growth of domains, and domain wall movements for *E* = 0 → *E*_max_ and *E* = *E*_max_ → 0 directions in the *P*−*E* measurement protocol are different from each other. This leads to a non-zero value of polarization, even at *E* = 0, known as remanent polarization (*P*_r_). As a result, a part of the stored energy is lost, which appears as the hysteresis of the *P*−*E* loop. In other words, it is impossible to recover the stored energy density to its fullest amount when it is discharged. The loss part of the stored energy or energy loss density (*U*_loss_) is given by the area of the loop. The recoverable energy density is calculated as follows:

(7) $$U_{rec}=\overset{P_{max}}{\underset{P_{r}}{\int }}EdP\;$$

Another FOM signifying the energy storage performance is the efficiency (*η*), which represents the amount of stored energy density available for use as recoverable energy density. It is defined as the ratio of the recoverable energy density to the total stored energy density:

(8) $$\eta =\frac{U_{rec}}{U_{st}}\times 100\%=\frac{U_{rec}}{U_{rec}+U_{loss}}\times 100\%$$

Equation (7) suggests that a combination of high *P*_max_, low *P*_r,_ and high breakdown strength are necessary to obtain a high *U*_rec_ value. In addition, Equation (8) requires a dielectric with low hysteresis loss to obtain a large efficiency value. Therefore, Equations (7) and (8) are often considered the governing equations for designing dielectric materials for high-performance energy storage. However, a dielectric with high *ε* usually features high dielectric loss, leading to heat generation during electric field cycling and the possibility of thermal breakdown during operation.

Typical *P*–*E* loops of LDs, FEs, AFEs, and RFEs are shown in Figure 2. LDs are characterized by very low values of polarization and a high BDS. Some of the widely studied LDs are CaTiO_3_ [19,20], SrTiO_3_ [21], and CaTiO_3_-CaHfO_3_ [22]. Because of the low value of polarization, the recoverable energy density of LD is quite low. Therefore, LDs are not suitable for application in the field of high-energy storage application. Over the years, FE and anti-ferroelectric (AFE) materials have been extensively studied for application in energy storage systems, and efforts to enhance their performance have surged. FE materials exhibit spontaneous polarization, a large value of *P*_max_, and a coercive field (*E*_c_). A typical *P*–*E* loop of FE materials is shown in Figure 2b. On the microscopic scale, FEs are composed of a large number of domains separated by domain walls. The dipoles in a domain are oriented in the same direction, and the directions of the domain polarizations can be switched by applying an electric field. However, the energy loss density is quite high in the FEs because of their high coercivity. Moreover, the *P*_r_ and *P*_max_ values have the same order of magnitude, resulting in a very small value of *P*_max_−*P*_r_. Therefore, the *U*_rec_ and *η* of the FEs are not promising. Because of this, single-phase FEs have not gained much interest in energy storage devices. Among the various FEs, representative compositions studied for energy storage are based on (Bi,Na)TiO_3_ [23,24,25,26], Ba(Zr,Ti)O_3_ [27,28,29], BaTiO_3_ [30,31], and (K,Na)NbO_3_ [32]. Unlike FEs, AFE materials lack a net polarization because of the anti-parallel alignment of the spontaneous polarization vectors in their domain. The typical *P*−*E* loops of AFE materials are shown in Figure 2c. The electric dipoles align anti-parallel to each other in the AFE domain, as shown in the inset of Figure 2c. At a low electric field, the polarization of the AFE materials varies linearly with the applied field. At a sufficiently high electric field, the electric dipoles in a domain rotate to align in the parallel direction, and the AFE behaves similarly to an FE with a further increase in the field strength. This is known as the field-induced AFE-FE transition. Once the electric field is removed, the induced FE phase reverts to the AFE state, thereby producing double hysteresis in the *P*−*E* loop. The high electric field for the AFE-FE phase transition (*E*_AFE−FE_) coupled with the high *P*_max_ and low *P*_r_ indicates the possibility of achieving high storage capacity in AFE materials. The most intensively studied AFE systems are based on (Pb, Zr)O_3_ [33,34,35,36], (Bi, Na)TiO_3_ [37,38,39], and AgNbO_3_ [40,41,42]. However, the *E*_AFE−FE_ for some AFEs is higher than their BDS at room temperature, signifying a breakdown before the transition to the highly polarized FE phase. Moreover, AFE materials cannot withstand large charge-discharge cycles, which is an important aspect in practical operations because such cycling leads the materials to undergo several alternate AFE-FE transitions, leading to physical cracks [43]. Moreover, the high-field FE phase often suffers from severe energy loss, which is particularly observed in AgNbO_3_-based AFEs [44].

## 3. Relaxor Ferroelectrics

Relaxor ferroelectrics (RFEs) are an important class of materials that have attracted significant interest in energy storage applications. RFEs exhibit nanosized polar regions embedded in a nonpolar matrix. The polar nanoregions (PNRs) exhibit spontaneous polarization; however, the inter-PNR interaction is very weak [45]. The typical size of a PNR is 2–10 nm. PNRs are highly dynamic and sensitive to external stimuli. Because of the lack of inter-PNR interactions, PNRs under an electric field evolve independently of nearby PNRs. Therefore, the polarization state can return to its initial state after the electric field is removed. The *P*−*E* loop for a typical RFE is shown in Figure 2d, which features *P*_r_ ~ 0, a considerably high *P*_max_, and a small hysteresis loop.

RFEs feature (i) a broad maximum in *ε* around *T*_m_ (maximum temperature in the *ε*-*T* curve); (ii) strong frequency dispersion of *ε* and loss tangent (tan *δ*) peaks, i.e., shifting of the peaks toward higher temperatures while measured at lower to higher frequencies; and (iii) low *P*_r_ [46,47]. Therefore, RFEs are FE materials that simultaneously exhibit dielectric relaxation and ferroelectricity. However, unlike normal FEs, where the paraelectric-FE phase transition can be explained by Curie’s law, the temperature dependence of *χ* in RFEs in the paraelectric phase obeys the following:

(9) $$\chi =\frac{C}{{\left(T-T_{c}\right)}^{\gamma }}$$

where the parameter *γ* (1 < *γ* < 2) represents the broadness of the dielectric peak. For a normal FE, *γ* = 1. Several models have been proposed to explain the peculiar characteristics of RFEs, such as the diffuse phase transition model [48], super paraelectric model [49], dipolar glass model [50], random-field model [51], random-site model [52], bi-relaxation model [53], and spherical random-bond-random-field model [54]. However, the underlying mechanism of RFEs is yet to be clearly understood.

## 4. Energy Storage Performance of BiFeO_3_-Based Relaxor Ferroelectrics

**Figure 3:**
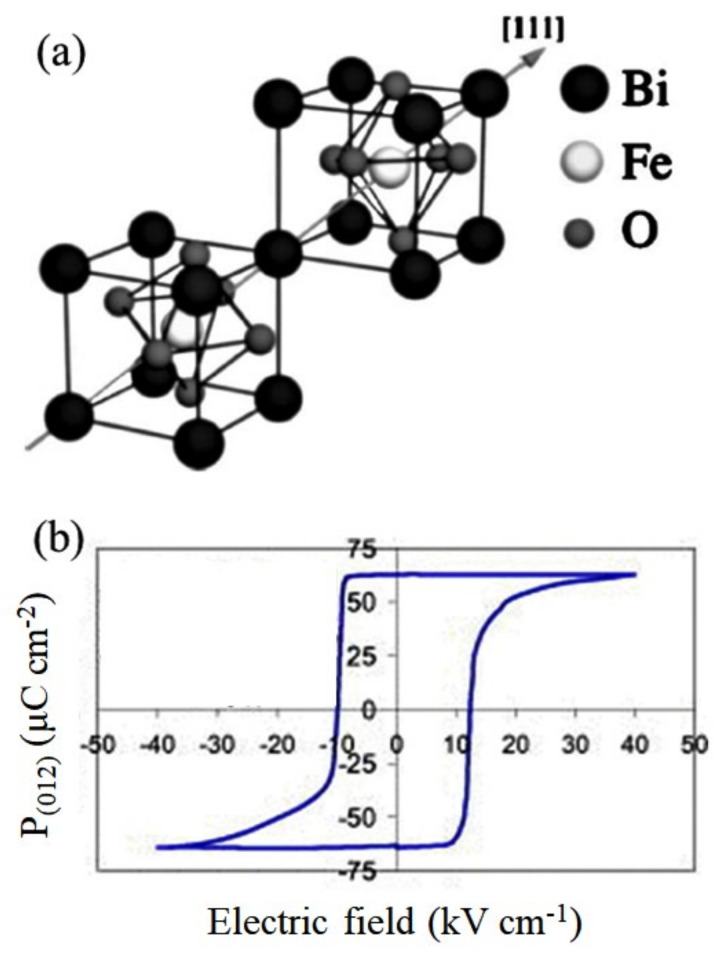
(**a**) Structure of R3c BiFeO_3_. (**b**) P-E loop of BiFeO_3_ bulk single crystal. Figures reproduced with permission from [55] and [61], respectively. © 2021 American Physical Society (**a**) and 2007 American Institute of Physics (**b**).

BiFeO_3_ exhibits a distorted perovskite structure, as shown in Figure 3a. It possesses a rhombohedral structure (point group: R3c) at room temperature with an *aˉaˉaˉ* tilt system, in which the neighboring oxygen octahedra rotate anti-clockwise about the [111] direction [55,56]. The rhombohedral unit cell is described with lattice constants *a* = *b* = *c* = 3.965 Å and *α* = *β* = *γ* = 89.3–89.4° [57]. There are two formula units of BiFeO_3_ in the rhombohedral cell, with three atoms in its asymmetric unit occupying Wyckoff positions: 6*a* (Bi^3+^ and Fe^3+^) and 18*b* (O^2−^) [58]. The unit cell can also be described in a hexagonal frame of reference with the hexagonal *c*-axis parallel to the diagonals of the cubic perovskite with the lattice constants *a*_hex_ = 5.58 Å and *c* = 13.90 Å [57].

The *R3c* symmetry permits long-range FE order in BiFeO_3_ along the threefold axis [111]. Various experiments have confirmed the ferroelectricity in BiFeO_3_ below *T*_C_ = 1143 K [55,59]. The constituent atoms Bi, Fe, and O are displaced from their centrosymmetric positions along the threefold axis, and Bi ions have the largest displacement with respect to O ions [55]. The lone-pair-active Bi ions in BiFeO_3_ are displaced to a large extent in comparison with other FE compounds with non-lone-pair-active cations. Therefore, a large value of spontaneous polarization, on the order of 90 μC cm^−2^, has been predicted in BiFeO_3_ from ab initio calculations [16,60]. However, a polarization value close to the calculated values could not be obtained until recently [61], after a series of initial experimental failures to achieve spontaneous polarization of BiFeO_3_, as predicted by theory [62,63,64,65,66,67]. Lebeugle et al. [61] measured a very large saturated polarization (approximately 60 μC cm^−2^) in a high-quality single-crystal BiFeO_3_ at room temperature, as shown in Figure 3b.

BiFeO_3_ possesses the highest values of spontaneous polarization and *T*_C_ among Pb-free FEs. As discussed in Section 2, high *P*_max_ and large *P*_max_ –*P*_r_ are among the most important factors for obtaining a high storage capacity. Therefore, BiFeO_3_ with a large *P*_r_ in its naturally occurring FE phase is not suitable for energy storage applications, which is a drawback of all FE materials in general. One way to obtain a small *P*_r_ is to break the long-range FE order such that it becomes an RFE. Many researchers have reported this method to create PNRs embedded in a non-FE matrix and obtain a significant reduction in the *P*_r_ value. In addition, there are always issues pertaining to the leakage current in pure BiFeO_3_, which eventually limits its high breakdown strength. Various successful methods to enhance the resistivity in phase-pure BiFeO_3_, such as doping at the A-site and the addition of Mn, have been discussed in the literature. In the following sections, considering these issues, the energy storage performance of BiFeO_3_-based materials, with special emphasis on the RFEs, are reviewed.

### 4.1. BiFeO_3_-Based Binary System

**Figure 4:**
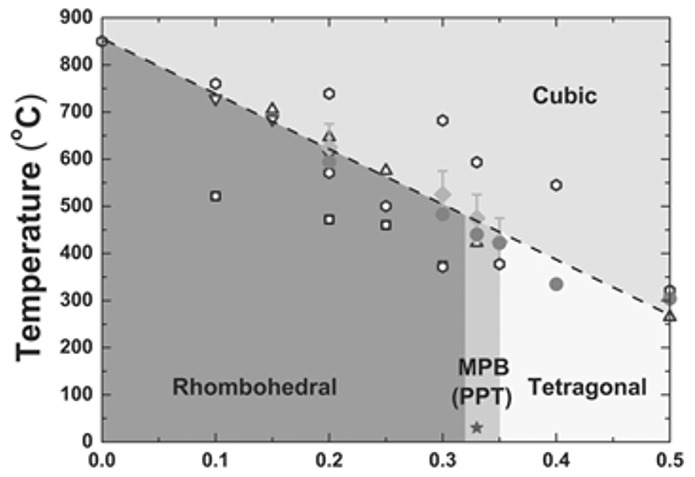
Phase diagram of (1-x)BiFeO_3_-xBaTiO_3_ solid solution. Reused with permission from [68]. © 2021 Willey-VCH Verlag GmbH & Co.

BiFeO_3_−BaTiO_3_ solid solutions have been widely investigated as promising candidates in the field of ceramic dielectrics-based energy storage materials. A remarkable feature of BiFeO_3_-based solid solutions is the morphotropic phase boundary (MPB), where the solid solution displays a composition-driven structural transition in its phase diagram. The crystal structure changes abruptly across the MPB, and various physical properties, such as piezoelectric coefficients and polarization, are maximal at the MPB. The MPB of the BiFeO_3_−BaTiO_3_ system is shown in the phase diagram in Figure 4, where the rhombohedral and tetragonal phases coexist in the MPB region [68,69,70]. Careful optimization of the BaTiO_3_ content produces excellent ferroelectric and piezoelectric properties in MPB compositions.

**Figure 5:**
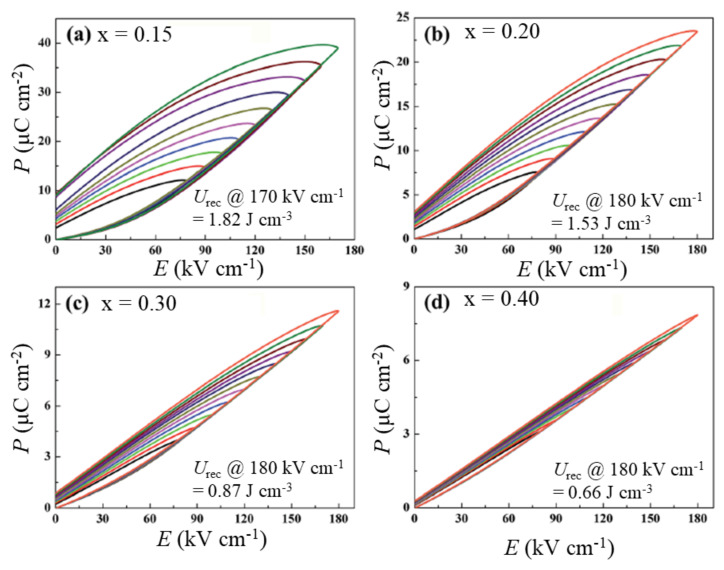
Unipolar P-E loops for 0.75Bi_1-x_Nd_x_FeO_3_-0.25BaTiO_3_ + 0.1 wt% MnO_2_ system: (**a**) x = 0.15, (**b**) x = 0.20, (**c**) x = 0.30, and (**d**) x = 0.40. Reproduced with permission from [76]. © 2021 The Royal Society of Chemistry.

However, BiFeO_3_-BaTiO_3_ also possesses a high *P*_r_ [71] and high dielectric loss, which are detrimental for energy storage. Previous studies have shown that doping small amounts of La_2_O_3_, MnO_2_, and Nb_2_O_5_ in BiFeO_3_ can significantly enhance the electrical resistivity and energy loss density [72,73], which is beneficial for energy storage. Wang et al. [74] reported a large enhancement of resistivity in Nb_2_O_5_-modified BiFeO_3_-BaTiO_3,_ i.e., (1-x)(0.65BiFeO_3_-0.35BaTiO_3_)–xNb_2_O_5_ (x = 0, 1, 3, 5 mol%) by several orders (~10^10^–10^14^ Ω cm) compared with the undoped BiFeO_3_-BaTiO_3_ (~10^8^ Ω cm). The compounds with x = 0.01 and 0.03 exhibited slimmer *P*−*E* loops, similar to RFEs, with x = 0.03 exhibiting the highest *P*_max_ (25.21 μC cm^−2^) and lowest *P*_r_ (5.53 μC cm^−2^). They obtained a maximum *U*_rec_ of 0.71 J cm^−3^ at *E* = 90 kV cm^-1^. Zhu et al. [75] found a significant improvement in the BDS of 0.52BiFeO_3_-0.48BaTiO_3_ ceramic up to 130–140 kV cm^−1^ by adding La_2_O_3_ and MnO_2_. Under this condition, they obtained *U*_rec_ = 1.22 J cm^−3^ with *η* = 58% for 0.52Bi_0.98_La_0.02_FeO_3_-0.48BaTiO_3_ + 0.3 wt.% MnO_2_ compound, whereas undoped 0.52BiFeO_3_-0.48BaTiO_3_ exhibited *U*_rec_ = 1.08 J cm^−3^ with *η* = 49%. They found that the addition of La_2_O_3_ and MnO_2_ increased the amount of the FE phase, reduced the grain size, and facilitated densification, which helped to induce large ∆*P* (*P*_max_ − *P*_r_) as well as BDS compared with undoped compounds. This phenomenon was found to be more pronounced when Nd was substituted for Bi sites in MnO_2_-added BiFeO_3_-BaTiO_3_ solid solution, as shown in Figure 5. Wang et al. [76] synthesized highly dense (relative density *ρ*_r_ = 95% to 97.6%) 0.75BiFeO_3_-0.25BaTiO_3_ ceramics by Nd substitution with 0.1 wt.% MnO_2_ addition that could endure a high electric field up to 180 kV cm^−1^, as shown in Figure 5. The solid solution with 15 mol% Nd content featured the highest value of *U*_st_ = 4.1 J/cm^3^ and *U*_rec_ = 1.82 J cm^−3^; however, it had a low value of *η* = 41.3%. Recently, Chen et al. [77] successfully synthesized highly dense Sm-doped BiFeO_3_-BaTiO_3_ binary ceramics that can endure a very high electric field of up to 200 kV cm^−1^. Sm substitution significantly reduced the grain size and enhanced the density, which is believed to be the reason for the high BDS. The binary solid solutions exhibit excellent *U*_st_ and *U*_rec_; however, their low efficiency limits practical device applications.

### 4.2. BiFeO_3_-Based Ternary System

#### 4.2.1. Bulk Ceramics

**Figure 6:**
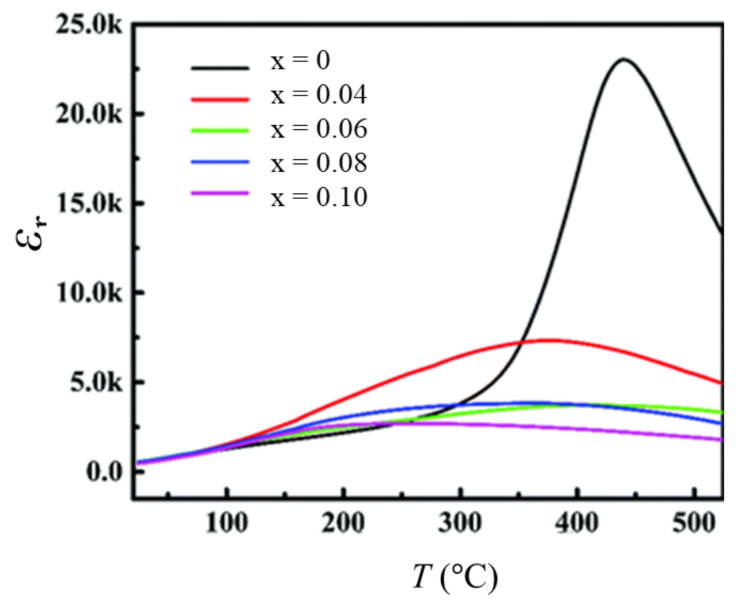
Induction of relaxor phase characterized by diffused phase transition in (0.66-x)BiFeO_3_-0.34BaTiO_3_-xBaZn_1/3_Ta_2/3_O_3_ systems. Reproduced with permission from [80]. © 2021 The Royal Society of Chemistry.

**Figure 7:**
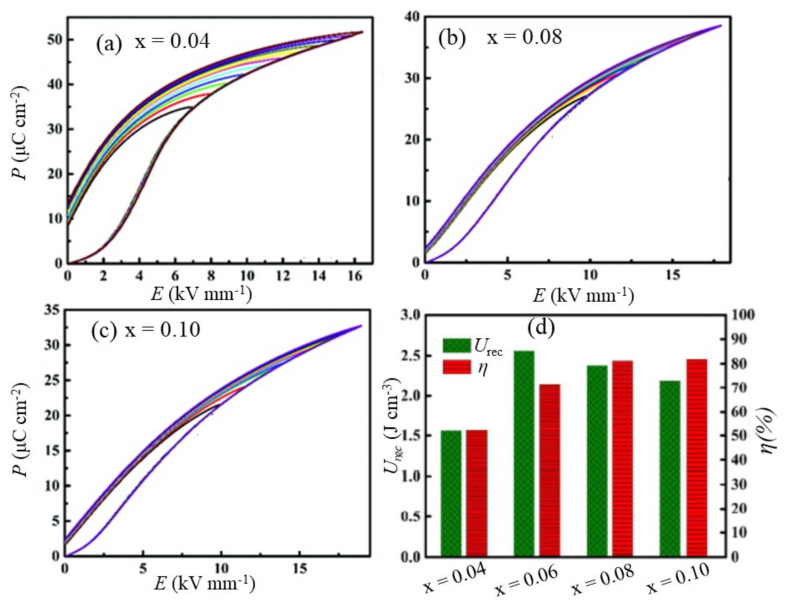
BaZn_1/3_Ta_2/3_O_3_ modification to BiFeO_3_-BaTiO_3_ leads to slimmer *P*–*E* loops. Unipolar *P*–*E* loops for (0.66-x)BiFeO_3_-0.34BaTiO_3_-xBaZn_1/3_Ta_2/3_O_3_ with (**a**) x = 0.04, (**b**) x = 0.08, and (**c**) x = 0.10. (**d**) The optimum recoverable energy density and efficiencies. Reproduced with permission from [80]. © 2021 The Royal Society of Chemistry.

The addition of a third perovskite oxide to binary BiFeO_3_-MTiO_3_ (M = Ba and/or Sr) as the end member of the ternary system has been found to be very promising for inducing the relaxor phase and enhancing the energy storage performance [78,79,80,81,82,83,84]. Zheng et al. [78] reported the successful induction of the relaxor phase in BiFeO_3_-BaTiO_3_ as a result of the substitution of BaMg_1/3_Nb_2/3_O_3_. The relaxor 0.61BiFeO_3_-0.33BaTiO_3_-0.06BaMg_1/3_Nb_2/3_O_3_ exhibited a *U*_rec_ of 1.56 J cm^−3^ at *E* = 125 kV cm^−1^, with *η* ~ 75%. This compound also exhibited good temperature stability for energy storage and efficiency in the temperature range of 25 °C to 190 °C. In a similar effort, Zheng et al. [79] reported an improved storage performance with a *U*_rec_ of 1.66 J cm^−3^ at 130 kV cm^−1^ and *η* ~ 82% in highly dense (*ρ*_r_ > 97%) 0.61BiFeO_3_-0.33BaTiO_3_-0.06LaMg_1/2_Ti_1/2_O_3_ ceramics. Meanwhile, Liu et al. [80] reported enhancement of relaxor characteristics in terms of broader peaks of dielectric permittivity (Figure 6) and significant enhancement of energy storage performance in (0.66 − x)BiFeO_3_-0.34BaTiO_3_-xBaZn_1/3_Ta_2/3_O_3_ for x > 0. They reported slim *P*–*E* loops for x > 0 with the highest BDS of *E* = 160 kV cm^−1^ and a high *U*_rec_ of 2.56 J cm^−3^ for the x = 0.06 composition (Figure 7). Tang et al. [84] reported a BDS of 180 kV cm^−1^ and *U*_rec_ = 1.62 J cm^−3^ for 0.85(0.65BiFeO_3_-0.35BaTiO_3_)-0.15Ba(Zn_1/3_Nb_2/3_)O_3_ bulk ceramics. Sun et al. [85] reported similar values of *U*_rec_ = 2.11 J cm^−3^ at *E* = 195 kV cm^−1^ with *η* = 84% in a highly dense 0.56BiFeO_3_-0.30BaTiO_3_-0.14AgNbO_3_+5 mol% CuO system prepared by a modified thermal quenching technique.

**Figure 8:**
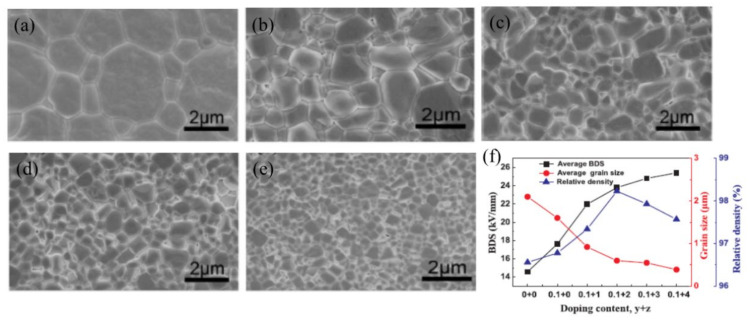
SEM images of 0.61BiFeO_3_-0.33(Ba_0.8_Sr_0.2_)TiO_3_-0.06La(Mg_2/3_Nb_1/3_)O_3_ + y wt.% MnO_2_ + z wt.% BaCu(B_2_O_5_) solid solution sintered at their optimum temperatures: (**a**) y = z = 0, (**b**) y = 0.1 and z = 1, (**c**) y = 0.1 and z = 2, (**d**) y = 0.1 and z = 3, and (**e**) y = 0.1 and z = 4. (**f**) Variations of BDS, grain size, and relative density with y + z. Reused with permission from [87]. © 2021 Elsevier Ltd.

Yu et al. [86] studied the effect of the microstructure on the energy storage performance of a BiFeO_3_-BaTiO_3_-Bi(Mg_2/3_Nb_1/3_)O_3_ system. They prepared coarse-grained (grain size ~2 to 4 μm) and fine-grained (~0.55 to 0.9 μm) microstructures using planetary ball milling and high-energy ball milling processes, respectively. BiFeO_3_-BaTiO_3_-Bi(Mg_2/3_Nb_1/3_)O_3_ solid solutions with a fine-grained microstructure exhibited higher ∆*P* (~ 30 μC cm^−2^) and BDS (~ 110 kV cm^−1^) than the coarse-grained samples (∆*P* ~ 10 μC cm^−2^, BDS ~ 50 kV cm^−1^). Under such drastic microstructural evolution, they reported a higher *U*_rec_ of 1.26 J cm^−3^ in fine-grained samples, compared with *U*_rec_ = 0.16 J cm^−3^ for coarse-grained samples. Yang et al. [87] showed that utilizing a liquid-phase sintering mechanism can significantly enhance the BDS while maintaining the relaxor characteristics and high dielectric permittivity. Using 2 wt% BaCu(B_2_O_5_) (BCB) as the low melting point additive in 0.1 wt% MnO_2_-added (0.67−x)BiFeO_3_–0.33(Ba_0.8_Sr_0.2_)TiO_3_-xLa(Mg_2/3_Nb_1/3_)O_3_ solid solution, they achieved a BDS of 230 kV cm^−1^ for x = 0.06. With the use of such a high field, the compound exhibited *U*_rec_ = 3.38 J cm^−3^ with *η* = 59%. BCB formed large amounts of liquid phase at the grain boundaries during sintering and significantly reduced the average grain size down to the submicron range, as shown in Figure 8, by impeding grain growth at lower sintering temperatures. The high density of grain boundaries in the microstructure of the submicron grain size offered high electrical resistivity, resulting in enhanced BDS (Figure 8). Moreover, low-temperature sintering with the addition of MnO_2_ helped to decrease Fe^3+^ ↔ Fe^2+^ valence fluctuations by minimizing Bi_2_O_3_ loss during synthesis, which was also critical for enhancing the BDS. This compound exhibited good temperature stability, with *U*_rec_ = 1.15–1.27 J cm^−3^ in the temperature range of 30 °C to 170 °C. Using the PVA-assisted viscous polymer process (VPP) route, Liu et al. [88] obtained high *ρ*_r_ ~ 99% 15 mol% Sr_0.7_Bi_0.3_FeO_3_–modified 0.85(0.65BiFeO_3_-0.35BaTiO_3_) system with a fine grain microstructure. The ultra-high *ρ*_r_ and uniform submicron grains significantly enhanced the BDS, with a value of 330 kV cm^−1^, compared with that of the sample prepared without VPP (180 kV cm^−1^). Under this condition, they obtain an ultra-high *U*_rec_ of 4.95 J cm^−3^ with *η* ~ 73%. The calculated *U*_rec_ from the charge-discharge cycling test was 2.36 J cm^−3^ at 300 kV cm^−1^. Likewise, Sm doping has been found to be very effective in reducing the grain size and increasing the density of sintered ceramics. Chen et al. [78] reported a significant decrease in the average grain size in the 0.67Bi_1_-_x_Sm_x_FeO_3_-0.33BaTiO_3_ system with an increase in x, resulting in a BDS of 200 kV cm^−1^ at x = 0.1. This resulted in an *U*_rec_ = 2.8 J cm^−3^ for x = 0.1; however, it had low efficiency, *η* = 55.8%. In another report, Li et al. [89] reported an ultra-high *U*_rec_ = 3.2 J cm^−3^ at *E* = 206 kV cm^−1^ with high efficiency, *η* = 92%, in a highly dense (*ρ*_r_ ~ 98%) solid solution of (1−x)Bi_0.83_Sm_0.17_Fe_0.95_Sc_0.05_O_3_-x(0.85BaTiO_3_-0.15Bi(Mg_0.5_Zr_0.5_)O_3_) with x = 0.75. A similar *U*_rec_ ≈ 3.06 J cm^−3^ at *E* = 167 kV cm^−1^ with *η* ≈ 92% associated with the improvement of BDS has been reported in (1−x)BiFeO_3_-x(0.85BaTiO_3_-0.15Bi (Sn_0.5_Zn_0.5_)O_3_) with x = 0.65 [90].

**Figure 9:**
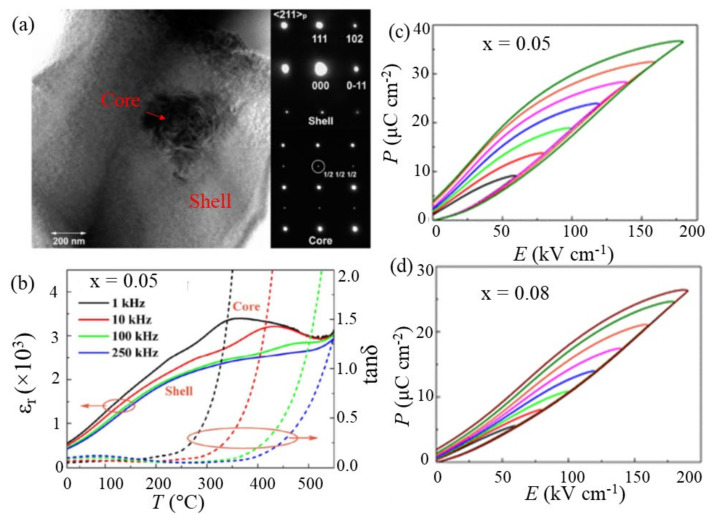
(**a**) Bright-field TEM image of a grain in (0.70-x)BiFeO_3_-0.30BaTiO_3_-xBi(Zn_2/3_Nb_1/3_)O_3_ with x = 0.05, illustrating a BiFeO_3_-rich core and BaTiO_3_-rich shell. (**b**) Broad dielectric anomalies associated with the core-shell structure. P-E loops for (**c**) x = 0.05 and (**d**) x = 0.08, respectively. Reproduced with permission from [81]. © 2021 American Chemical Society.

Designing a specific microstructure has been found to be highly effective for enhancing energy storage performance. Microstructure strongly influences the BDS of dielectric materials and their relaxor characteristics. Wang et al. [81] designed core-shell microstructure (Figure 9a) with BaTiO_3_-rich shells and BiFeO_3_-rich cores in (0.7-x)BiFeO_3_-0.30BaTiO_3_-xBi(Zn_2/3_Nb_1/3_)O_3_+0.1wt%Mn_2_O_3_ ceramics that could withstand an electric field as high as 190 kV cm^−1^. They found that the shells and cores in the solid solutions were different in structure and electrical characteristics. The shell exhibited a pseudo-cubic structure, which is paraelectric in nature, whereas the core parts had a ferroelectric R3c structure. The core-shell structure and the cationic charge disorder at the B-sites helped to establish the relaxor phase in 0.70BiFeO_3_-0.30BaTiO_3_ (Figure 9a). Substitution of Bi(Zn_2/3_Nb_1/3_)O_3_ further exacerbated the long-range order, thereby inducing a highly disordered RFE phase while maintaining the polarizability as high as *P*_max_ = 36.7 μC cm^−2^ and ∆*P* = 32.8 μC cm^−2^. They obtained *U*_st_ = 3.7 J cm^−3^, *U*_rec_ = 2.06 J cm^−3^ at 180 kV cm^−1^ and *U*_st_ = 2.9 J cm^−3^, *U*_rec_ = 1.98 J cm^−3^ at 190 kV cm^−1^ for 5 mol% and 8 mol% Bi(Zn_2/3_Nb_1/3_)O_3_-doped compounds, respectively. The solid solutions could also successfully deliver discharge energy within 0.5 μs. Wang et al. [82] reported relaxor behavior in chemically inhomogeneous, but electrically homogeneous (0.7-x)BiFeO_3_-0.3BaTiO_3_-xNd(Zr_0.5_Zn_0.5_)O_3_ for x = 0.05, 0.08, and 0.10, with a core-shell structure, and studied the energy storage performance at room temperature both in ceramics and multilayers prepared by a solid-state reaction route. The substitution of Nd(Zr_0.5_Zn_0.5_)O_3_ induced chemical inhomogeneity, revealed as a (Bi, Fe)-rich core for x = 0.05 and (Ba,Ti)-rich cores for x = 0.1, and complex multiphase microstructures with both (Bi, Fe)-rich and (Ba,Ti)-rich cores for x = 0.08. A high degree of chemical inhomogeneity and better electrical homogeneity of grains existed at x = 0.08, which led to a high ∆*P* and provided a difficult current path for electrical breakdown. As a result, *x* = 0.08 exhibited *U*_rec_ ~2.45 J cm^−3^ at *E* = 240 kV cm^−1^ with *η* = 72%. Lu et al. [91] demonstrated excellent energy storage properties in a series of solid solutions composed of BiFeO_3_, SrTiO_3_, Nb_2_O_5_, and BiMg_2/3_Nb_1/3_O_3_ exhibiting a core-shell structure. Their study successfully demonstrated that Nb_2_O_5_ doping into BiFeO_3_-SrTiO_3_ and employing the third perovskite end member BiMg_2/3_Nb_1/3_O_3_ (BMN) could induce an insulating relaxor phase at room temperature without reducing the average ionic polarizability of the solid solution. It was found that the substitution of 1–3% Nb^5+^ for Ti^4+^ (B sites) in 0.6BiFeO_3_-0.4SrTiO_3_ suppressed the formation of oxygen vacancies and significantly reduced *p*-type conductivity compared with that of the undoped compound. A similar reduction in the *p*-type conductivity was observed for the 0.56BiFeO_3_-0.4SrTiO_3_-0.04BiMg_2/3_Nb_1/3_O_3_-xNb_2_O_5_ (x = 0–0.05) solid solution, and an enhanced BDS of 360 kV cm^-1^ was obtained at an optimized Nb_2_O_5_ content (x = 0.03). Such a high BDS was attributed to two factors: (i) the improved insulating character caused by Nb doping at B-sites related to the suppression of the formation of oxygen vacancies, and (ii) a core-shell microstructure with electrical homogeneity throughout the grains. The *x* = 0.03 composition exhibited a high performance of *U*_rec_ = 6 J cm^−3^ with *η* = 74.6%. Then, a much-improved *U*_rec_ value with similar *η* could be achieved by optimizing the BiMg_2/3_Nb_1/3_O_3_ content in (0.6-y)BiFeO_3_-0.4SrTiO_3_-0.03Nb_2_O_5_-yBiMg_2/3_Nb_1/3_O_3_ (y = 0.02–0.12) solid solutions. For y = 0.1, the BDS was enhanced further up to 460 kV cm^−1^, producing *U*_rec_ = 8.2 J cm^−3^ and *η* = 74.6%. A core-shell microstructure design provides a large BDS and large *U*_rec_, but at the same time, it has low efficiency because of the not-very-fast response of the PNRs to the electric field.

**Figure 10:**
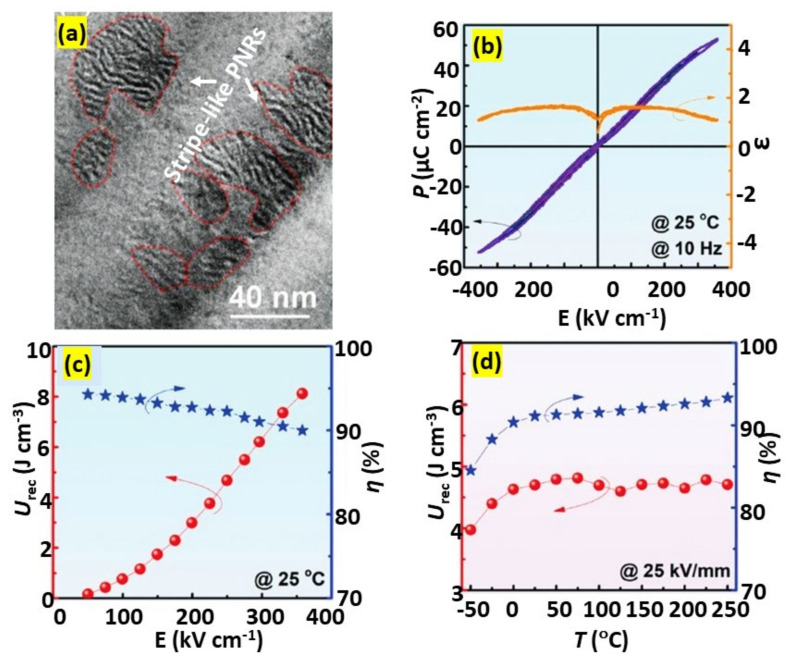
(**a**) Domain morphology exhibiting stripe-like PNRs in 0.57BiFeO_3_-0.33BaTiO_3_-0.1NaNbO_3_ with the addition of 0.1 wt.% MnO_2_ and 2 wt.% BaCu(B_2_O_5_). (**b**) Room-temperature *P*–*E* loops and *ε* measured under various electric fields for the same compound. *U*_rec_ and *η* measured at (**c**) various electric fields and (**d**) temperatures. Figures reused with permission from [92]. © 2021 WILEY-VCH Verlag GmbH & Co. KGaA, Weinheim.

Qi et al. [92] employed a domain engineering technique to optimize the microstructure at the domain level and showed that this technique was very effective for enhancing *U*_rec_ and *η*. Using it, they obtained large *P*_max_, large ∆*P*, and superior energy storage performance in 0.57BiFeO_3_-0.33BaTiO_3_-0.1NaNbO_3_ with the addition of 0.1 wt% MnO_2_ and 2 wt% BaCu(B_2_O_5_). Nanodomain engineering produced stripe-like PNRs embedded in featureless nanodomains. The stripe-like PNRs were rich in BiFeO_3_, whereas the matrix domains were rich in BaTiO_3_ and NaNbO_3_. An HR-TEM image of the solid solution is shown in Figure 10a. Stripe-like PNRs rich in BiFeO_3_ were dispersed in BaTiO_3_- and NaNbO_3_-rich featureless nanodomains. This structural heterogeneity at the domain level led to a rapid polarization response to the external *E*-field, unlike in the core-shell microstructure, and produced a hysteresis-free *P*−*E* loop, as well as a large *P*_max_. Moreover, NaNbO_3_ substitution increased the bandgap and helped to obtain a uniform and fine-grained microstructure, which was beneficial to enhance BDS up to 360 kV cm^−1^, producing *U*_rec_ ≈ 8.12 J cm^−3^ with *η* ≈ 90%. This solid solution also exhibited excellent thermal stability, with *U*_rec_ = 8.12 J cm^−3^ ± 10% in the temperature range −25 °C to 250 °C, and an ultrafast discharge rate (< 100 ns).

#### 4.2.2. Thin Films

In the past few decades, thin films have gained enormous scientific importance in a plethora of applications. The obvious advantages thin films possess over their bulk counterparts are that they save material and have reduced weight. In addition, it is well known that material properties drastically change when they are deposited in thin film forms, which paves the way for industrial applications. Dielectric thin films with thicknesses on the nano- or submicron scale have shown promising potential in the field of energy storage for low-power small devices. This is because of their extraordinarily high BDS (1 MV/cm) and energy density compared with bulk dielectrics.

Correia et al. [93] deposited a thin film of 0.4BiFeO_3_-0.6SrTiO_3_ onto a SrRuO_3_-electroded (100)-SrTiO_3_ substrate using the pulsed laser deposition (PLD) method, which can endure an electric field as large as 0.972 MV cm^−1^. Under such a high electric field, the film exhibited *U*_rec_ = 18.6 J cm^−3^ with *η* > 85%. It also featured a small temperature coefficient of capacitance (TCC < 11%) over a wide range of temperatures up to 200 °C. Pan et al. [94] demonstrated that the BDS of the same material could be enhanced more than by three times, up to 3.6 MV cm^−1^, when doped with Mn and deposited onto a Nb-doped SrTiO_3_ (001) substrate by the PLD technique. The high BDS of the Mn-doped 0.4BiFeO_3_-0.6SrTiO_3_ thin film was attributed to (i) a dense, uniform, and crack-free microstructure, (ii) high-quality epitaxial growth, and (iii) low leakage current by reducing the Fe^3+^/Fe^2+^ valence fluctuation with Mn substitution. They obtained a colossal *U*_rec_ = 51 J cm^−3^ and *η* = 64%, which are comparable to those of lead-based thin films (*U*_rec_ = 61 J cm^−3^ and *η* = 33%) [95]. The thin film also featured high fatigue endurance quality over 2 × 10^7^ cycles and good thermal stability over a wide range of temperatures, from −40 °C to 140 °C. In another report, Pan et al. [96] utilized a domain engineering method to obtain strong relaxor behavior in (1−x)BiFeO_3_-xSrTiO_3_ thin films (x = 0.3–0.75) deposited by the PLD technique and found that the BDS of the films could be enhanced further, up to 4.46 MV cm^−1^, by increasing the SrTiO_3_ content. Atomic-scale microstructure analysis based on the STEM of BiFeO_3_-SrTiO_3_ films revealed that SrTiO_3_ disrupted the long-range FE order, and this disruption cascaded with an increase in SrTiO_3_ content. The incorporation of SrTiO_3_ could transform the micrometer-scale FE domains into nanoscale PNRs. Paraelectric SrTiO_3_ acts as a matrix for embedded PNRs and separates the PNRs such that the inter-PNR interaction almost vanishes. Because there are no inter-PNR interactions, the PNRs are very dynamic under an external electric field and produce slim *P*–*E* loops with very small *P*_r_ values. The size of the PNRs continued to decrease with increasing SrTiO_3_ content, forming an almost domain-less feature for x = 0.75. This domain evolution reduced the domain switching energy, producing a slim *P*–*E* loop, while maintaining a large *P*_max_ value and a colossal BDS. In addition to the domain perspective, SrTiO_3_ substitution enhanced the insulating character of the films by stabilizing the Fe^3+^/Fe^2+^ valence fluctuation and reducing the formation of oxygen vacancies, leading to further enhancement of BDS. For example, the BDS value of (1-x)BiFeO_3_-xSrTiO_3_ films increased from 2.77 MV cm^−1^ to 4.46 MV cm^−1^ as the SrTiO_3_ content increased from for *x* = 0.3 to *x* = 0.75. At such a high electric field, the films with *x* = 0.6 and 0.75 exhibited a giant *U*_rec_ ~ 70 J cm^−3^.

**Figure 11:**
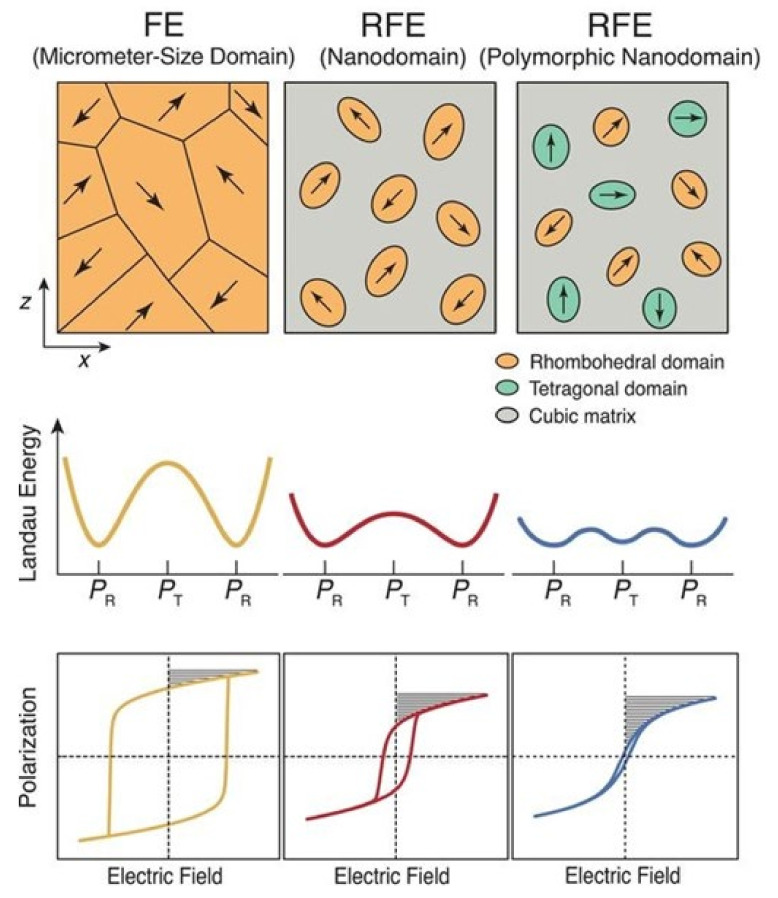
Design of RFEs via polymorphic nanodomain design. Comparative display of Landau energy profiles and *P*−*E* loops of an FE with micrometer-sized domains, an RFE with nanodomains, and an RFE with polymorphic nanodomains. The *P*_R_ represents the polarization states along the rhombohedral (*R*) directions, and *P*_T_ is along the tetragonal (*T*) direction. The shadowed area in the *P*-*E* loops represents the recoverable energy density. Figures reproduced with permission from [97]. © 2021 The Authors, some rights reserved; exclusive licensee American Association for the Advancement of Science.

**Figure 12:**
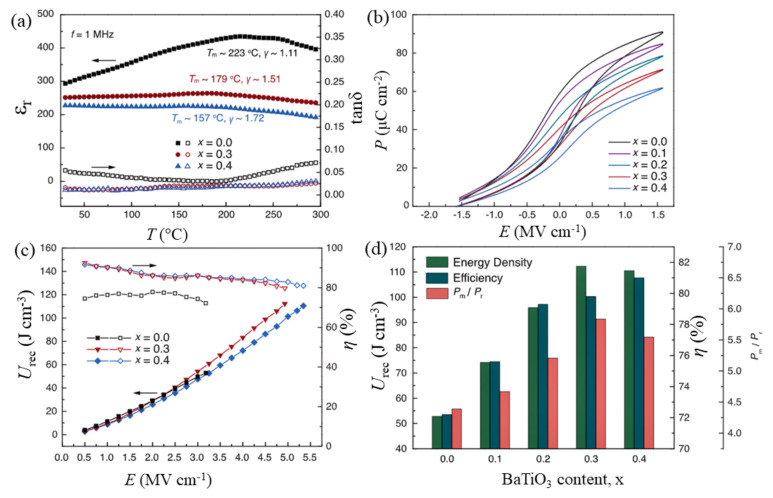
Characteristics of (0.55−*x*)BiFeO_3_-*x*BaTiO_3_-0.45SrTiO_3_ (x = 0.0–0.4) films. (**a**) Temperature-dependent dielectric permittivity and loss tangent at a frequency of 1 MHz, (**b**) first-order reversal curve (FORC) *P*−*E* loops, (**c**) energy density and efficiency values with respect to applied electric fields up to breakdown fields, and (**d**) comparison of the energy storage performance at breakdown fields. Figures reproduced with permission from [97]. © 2021 The Authors, some rights reserved; exclusive licensee American Association for the Advancement of Science.

Domain engineering techniques have been found to be more fruitful in thin films that exhibit polymorphic nanodomains, e.g., rhombohedral (*R*) and tetragonal (*T*) domains in a cubic paraelectric matrix. If these polymorphs have competitive free energy, Landau phenomenological theory predicts the weakening of polarization anisotropy and lowering of the energy barrier between the *R* and *T* polarization states [97]. It facilitates a flatter energy profile for polymorphic nanodomain RFEs compared with classic FEs and nanodomain RFEs, which minimizes the hysteresis while maintaining a high polarization (Figure 11). Pan et al. [97] demonstrated that the polymorphic domain engineering technique can produce ultra-high energy density with high efficiency in thin films of a (0.55−x)BiFeO_3_-xBaTiO_3_-0.45SrTiO_3_ solid solution (x = 0 to 0.4). Here, BiFeO_3_ and BaTiO_3_ were the hosts for the *R* and *T* domains, whereas SrTiO_3_ provided a cubic paraelectric matrix. The relaxor nature and energy storage performance of the (0.55−x)BiFeO_3_-xBaTiO_3_-0.45SrTiO_3_ solid solutions are shown in Figure 12. The incorporation of BaTiO_3_ gradually enhanced the relaxor nature, as can be seen from the wider peaks in the ε–T plots (Figure 12a), as well as the BDS for higher BaTiO_3_ contents. The BDS increased up to 4.9 MV cm^−1^ for x = 0.3 and 5.3 MV cm^−1^ for x = 0.4, compared with 3.2 MV cm^−1^ for the x = 0 compound. This resulted in a maximum *U*_rec_ of 112 J cm^−3^ and 110 J cm^−3^ for x = 0.3 and 0.4, respectively, with *η* > 80% (Figure 12). Moreover, the enhancement of the relaxor nature resulted in good temperature stability for x = 0.3 and 0.4 regarding their energy storage performance over a wide range of temperatures. Kurusumovic et al. [98] employed a combined defect engineering method to explore the energy storage performance of relaxor thin films of BiFeO_3_-BaTiO_3_ solid solutions doped with Mn. The combined approach of defect engineering consisted of an interval mono-layer by mono-layer deposition (LLD) and Mn addition. The LLD produced highly stoichiometric and perfectly crystalline films compared with standard deposited films, while the addition of Mn reduced the leakage current by creating vacancy trap centers. Using this method, they obtained an ultra-high value of *U*_rec_ = 80 J cm^−3^ at a BDS of 3.1 MV cm^−1^ with *η* = 78% in 2.5 mol.% Mn-doped 0.25BiFeO_3_-0.75BaTiO_3_ thin films.

### 4.3. Multilayered Structure

Although the energy density of thin films is superior to that of bulk ceramics, the usability of thin films is limited for low-power applications because of their small volume. In this context, multilayered structures have received great attention because the technology behind them is well known and inexpensive. A multilayered ceramic consists of a number of thin ceramic layers with thicknesses on the micrometer scale and internal electrode layers stacked in parallel and connected through terminal electrodes. It features both high BDS (on the MV cm^−1^ level) and large volume; therefore, it is very promising for practical high-power energy storage applications.

**Figure 13:**
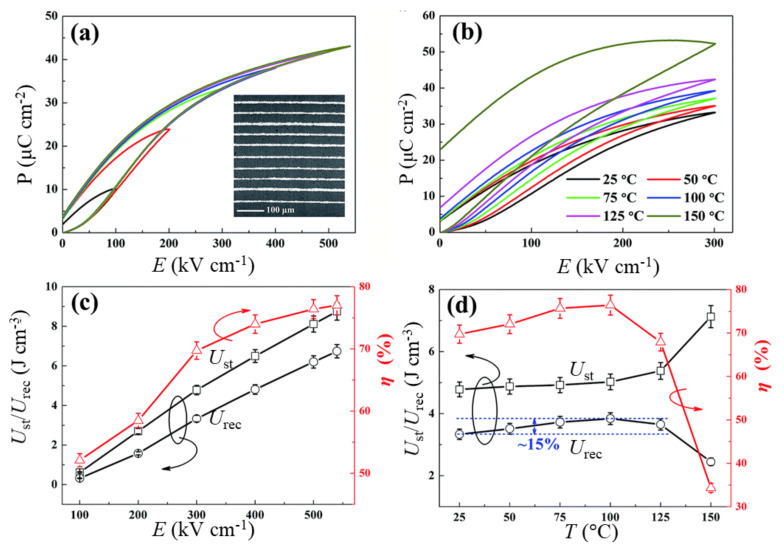
Energy storage performance of 0.75(Bi_0.85_Nd_0.15_)FeO_3_-0.25BaTiO_3_ multilayers. (**a**) P–E loops at various electric fields at room temperature, (**b**) P–E loops at various temperatures. *U*_st_, *U*_rec_, and *η* with (**c**) electric field and (**d**) temperature. An SEM image of the multilayers is shown in the inset of (**a**). Figures reproduced with permission from [76]. © 2021 The Royal Society of Chemistry.

**Figure 14:**
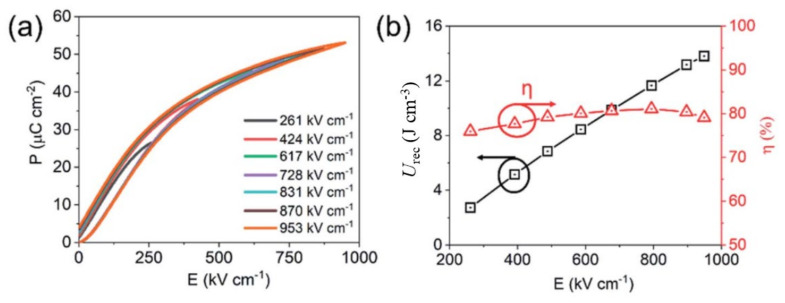
(**a**) Room temperature unipolar *P*–*E* loops at various electric fields and (**b**) calculated energy storage properties of 0.57BiFeO_3_-0.3BaTiO_3_-0.13Bi(Li_0.5_Nb_0.5_)O_3_ multilayers. Figures reused under Creative Commons Attribution 3.0 Unported Licence [101]. © 2021 The Royal Society of Chemistry.

Wang et al. [76] reported a large improvement in the energy storage performance of a multilayered ceramic composed of 0.75(Bi_0.85_Nd_0.15_FeO_3_)-0.25BaTiO_3_ + 0.1wt.% MnO_2_ and Pt internal electrodes, compared with its bulk ceramic counterpart, as shown in Figure 13. With nine active layers having a total thickness of ~ 0.78 mm, they obtained a significant enhancement of the BDS to 540 kV cm^−1^ with *U*_st_ ~ 8.75 J cm^−3^, *U*_rec_~ 6.74 J cm^−3^, and *η*~77%. Yan et al. [99] reported similar energy storage properties in (1-x)(0.67BiFeO_3_-0.33BaTiO_3_)-xNa_0.73_Bi_0.09_NbO_3_ multilayered ceramics with A-site cation vacancies. For an optimized content of Na_0.73_Bi_0.09_NbO_3_, i.e., x = 0.12, the multilayer exhibited *U*_rec_ = 5.57 J cm^−3^ at *E* = 410 kV cm^−1^ with *η* = 83.8%. A multilayer ceramic with 7-μm-thick 0.61BiFeO_3_-0.33Ba_0.8_Sr_0.02_TiO_3_-0.06La(Mg_2/3_Nb_1/3_)O_3_ dielectric layers was reported to exhibit a much higher BDS, > 740 kV cm^−1^ [100]. This device featured a high *U*_rec_ of 10 J cm^−3^ with *η* ~ 72% at *E* = 730 kV cm^−1^. Wang et al. reported a large ∆*P* of ~ 34 μC cm^−2^ and a high BDS of 700 kV cm^−1^ in a multilayered ceramic composed of 16-μm-thick electrically homogeneous 0.62BiFeO_3_-0.3BaTiO_3_-0.08NdZr_1/2_Zn_1/2_O_3_ ceramic layers with a core-shell microstructure [82]. With seven ceramic layers having an active electrode area of 33 mm^2^, *U*_rec_ as high as 10.5 J cm^−3^ with *η* = 87% was obtained. Lu et al. [91] obtained BDS > 1 MV cm^−1^ in a multilayered ceramic with 10 mol% Bi(Mg_2/3_Nb_1/3_)O_3_ and 3 mol% Nb_2_O_5_-doped 0.6BiFeO_3_-0.4SrTiO_3_ with a core-shell microstructure. The device, with 8-μm-thick dielectric layers, exhibited *U*_rec_ = 15.8 J cm^−3^ under *E* > 1 MV cm^−1^ with *η* = 75.2%. Wang et al. [101] reported a high BDS of 953 kV cm^−1^ in a multilayered ceramic with 8-μm-thick 0.57BiFeO_3_-0.3BaTiO_3_-0.13Bi(Li_0.5_Nb_0.5_)O_3_ layers (Figure 14) having a core-shell structure and a 5-mm^2^ active electrode area, which was greatly improved compared with that of the bulk ceramic counterpart (260 kV cm^-1^). While the bulk ceramic featured a *U*_rec_ ~ 3.64 J cm^−3^ at *E* ~ 260 kV cm^−1^ with *η* ~ 75%, the multilayered ceramic exhibited a *U*_rec_ = 13.8 J cm^−3^ with *η* = 81% because of the large enhancement of the BDS. The multilayered ceramic showed good temperature stability (< 10%) and frequency independence (< 5%) of *U*_rec_ from 0.01 to 1 Hz, as well as fatigue resistance (< 5%) during 10^4^ cycles of unipolar *P*−*E* loop tests in the temperature range from RT to 100 °C at *E* = 400 kV-cm^−1^.

## 5. Critical Issues and Strategy

### 5.1. Leakage Current Control

High discharge energy density and energy efficiency are the primary requirements of an EES system. However, most BiFeO_3_-based RFEs suffer from a low efficiency (<85%) despite having ultra-high discharge energy densities. Leakage-related high conductivity in BiFeO_3_-based RFEs produces apparent large values of *P*_r_ and wide hysteresis loops, which lead to a higher loss density. The conductivity usually increases at high temperatures, limiting their potential for high-temperature applications.

The high leakage current in BiFeO_3_-based compounds is associated with the loss of Bi_2_O_3_ during its synthesis at elevated temperatures (950–1050 °C), which creates ionic vacancies and Fe^3+^/Fe^2+^ valence fluctuation [102,103,104,105]. Aliovalent doping in BiFeO_3_-based compounds has been effective for reducing the leakage current density by several orders of magnitude [104,105]. However, these compounds are fired at high temperatures for synthesis in both bulk ceramics and multilayered structures, exposing them to Bi_2_O_3_ loss. Adopting a low-temperature sintering method is expected to reduce the Bi_2_O_3_ loss and the associated leakage current densities by a few more orders. The addition of appropriate glass additives and/or low melting point compounds is expected to significantly reduce the sintering temperature [106], thereby reducing the possibility of Bi_2_O_3_ volatilization. Low-temperature sintering is also beneficial for the enhancement of BDS, as discussed in Section 5.2.

### 5.2. Microstructure Engineering

**Figure 15:**
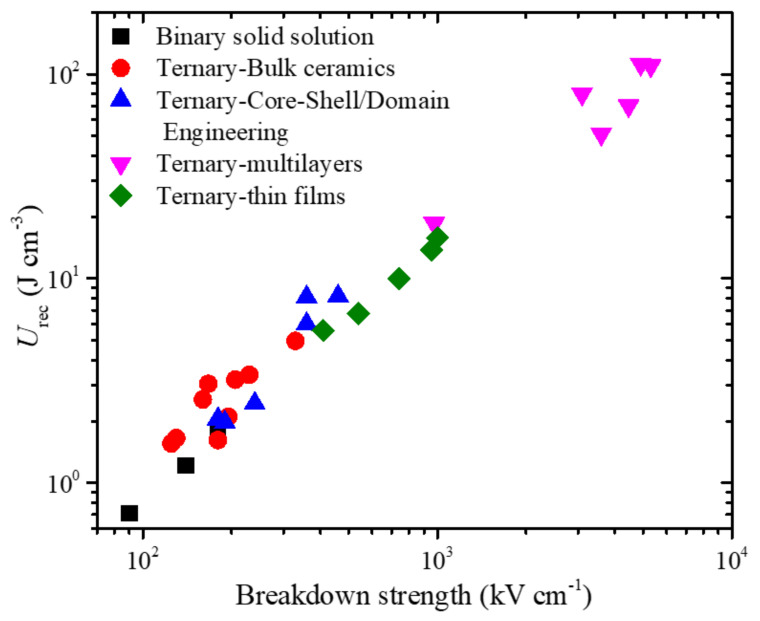
Recoverable energy density as a function of breakdown strength for BiFeO_3_-based bulk ceramics of binary solid solutions and bulk ceramics, microstructure-designed bulk ceramics, and multilayers and thin films of ternary solid (TS) solutions.

In Figure 15, we plot *U*_rec_ as a function of BDS for BiFeO_3_-based binary and ternary solid solutions fabricated in bulk ceramics, ceramic multilayers, and thin films. A high BDS usually provides a higher recoverable energy density. Therefore, enhancing the BDS is one of the most effective ways to increase the energy storage performance of RFEs. Microstructure engineering, such as the design of core-shell structures and domain engineering, has been found to be very effective for enhancing the BDS and realizing a dynamic relaxor ferroelectric phase. The BDS depends on the grain size (BDS ∝ 1/√*G* (*G* = average grain size)) and size distribution. Therefore, it is imperative to design a fine-grained microstructure with a uniform size distribution to obtain a high BDS. Another way to improve the BDS is to prepare a highly dense microstructure. Pores or cavities present in microstructures are usually filled with gaseous or liquid phases with lower permittivity than those of solid dielectrics. As a result, the voltage across the cavities or pores (*V*_c_) is enhanced as per the following relation [107]:

(10) $$V_{c}=\frac{V_{app}}{\left[1+\frac{\varepsilon_{c}}{\varepsilon_{r}}\left(\frac{d}{t}-1\right)\right]}$$

where *V*_app_ is the applied voltage, *ε*_c_ and *ε*_r_ are the permittivity of the cavity and dielectric, respectively, and *t* and *d* are the size of the cavity and the thickness of the dielectric, respectively. A small pore can create a large electric field across it and cause local breakdown and internal discharge even at a low external voltage. Therefore, a highly dense microstructure is essential. Because BiFeO_3_-based materials are prone to the loss of Bi and Fe valence fluctuations during sintering at high temperatures, resulting in high electrical conductivity, sintering at low temperatures could be very beneficial. However, a detailed investigation of the microstructure control for the purpose of enhancing the energy storage performance employing the low-temperature sintering technique in BiFeO_3_-based dielectrics is yet to be conducted. The two-step sintering (TSS) method modified by Chen and Wan [108] is one of the most cost-effective and simple methods to produce ultra-high-density materials and fine grains. In this technique, high-temperature heating is performed for a few minutes, and then the material is allowed to cool to the sintering temperature, where the sample is sintered for a prolonged time. However, the sintering temperature should be wisely chosen so that densification without further grain growth occurs.

### 5.3. Band Gap Engineering

Electronic breakdown is a crucial intrinsic mechanism for the breakdown of a dielectric in a large electric-field regime. Above a certain electric field, electrons in the valence band gain sufficient energy to jump to the conduction band. This results in an increase in the electron density in the conduction band and leads to a large current discharge. Therefore, an insulator exhibiting a wide forbidden energy gap can withstand a high electric field. The empirical relation between the BDS and energy band gap is given by the following relation [109]:

(11) $$E_{B}=24.442\;exp\left(0.315\sqrt{E_{g}\omega_{max}}\right)$$

where *E*_g_ is the energy bandgap, and *ω*_max_ is the maximum phonon frequency. Pure BiFeO_3_ has a direct bandgap of 3 eV [110]. Only a few studies have attempted to enhance the BDS of BiFeO_3_-based RFEs via modulation of the energy bandgap. Qi et al. [92] reported the enhancement of BDS by doping of high-band-gap materials such as NaNbO_3_ (*E*_g_ ~ 3.28 eV) in 0.67BiFeO_3_-0.33BaTiO_3_ solid solutions. They demonstrated that the bandgap of the solid solutions (0.67-x)BiFeO_3_-0.33BaTiO_3_-xNaNbO_3_ (0 ≤ x ≤ 0.15) increases monotonically with increases in x from ~ 2.6 eV for x = 0 to ~ 2.95 eV for x = 0.15. The enhancement of BDS followed a similar trend with an increase in NaNbO_3_ content, where the average BDS increased from 230 kV cm^−1^ for x = 0 to 420 kV cm^−1^ for x = 0.15. Further studies on bandgap engineering can be explored to enhance the BDS and energy storage performance of BiFeO_3_-based relaxor ferroelectrics.

### 5.4. Electromechanical Breakdown

Another issue that has not gained much attention but is very critical in BiFeO_3_-based relaxor ferroelectrics is the electromechanical breakdown. Because a high electric field is required to obtain a large recoverable energy density, the dielectrics are under extreme electrostrictive strain, increasing the mechanical breakdown. This issue should be taken very seriously from the device viewpoint, as the devices undergo a large number of charge-discharge cycles. Although high recoverable energy density with high efficiency is obtained at high electric fields under laboratory conditions, the same samples might not be suitable for application purposes where they totally collapse because of mechanical failure under a large number of charge-discharge cycles. However, systematic studies on the issues of electromechanical breakdown in BiFeO_3_-based dielectrics are lacking. Rare-earth-ion doped BiFeO_3_ compounds with their composition lying across the morphotropic phase boundary could be interesting in this regard as these compounds feature high electromechanical strain. For example, Walker et al. [111] reported cycle-dependent large electromechanical strain in Sm-doped BiFeO_3_ polycrystalline samples, associated with the electric field-induced phase transition and ferroelectric/ferroelastic domain switching.

## 6. Summary

BiFeO_3_-based relaxor ferroelectrics are projected to be potential Pb-free candidates for application in the field of high-energy-density storage and high-power-delivery systems. They are mostly fabricated in the form of ceramics, multilayers, and thin films from binary or ternary solid solutions with other perovskite oxides. BiFeO_3_-BaTiO_3_ and BiFeO_3_-SrTiO_3_, with their compositional ratios lying in the morphotropic phase boundary, are the two most widely studied BiFeO_3_-based binary solid solutions for high-energy-density storage. However, the typical value of recovering energy density of bulk ceramics of BiFeO_3_-based binary solid solutions is well below 3 J cm^−3^, with low efficiency (typically below 50%). Ternary solid solutions exhibiting specific microstructures, such as core-shell structures and stripe-like nanodomains, have shown significant enhancement of the energy density properties. For example, 0.5BiFeO_3_-0.4SrTiO_3_-0.03Nb_2_O_5_-0.1BiMg_2/3_Nb_1/3_O_3_ bulk ceramic with a core-shell microstructure exhibited excellent energy storage properties, with *U*_rec_ ~ 8.2 J cm^−3^ and *η* ~ 74%. The nanodomain-engineered bulk ceramic 0.57BiFeO_3_-0.33BaTiO_3_-0.1NaNbO_3_ with the addition of 0.1 wt% MnO_2_ and 2 wt% BaCu(B_2_O_5_) also featured a similar value of *U*_rec_ (~ 8.12 J cm^−3^); however, it had a higher value of *η* ~ 90%. The microstructure design approach has been found to be attractive for enhancing the energy storage properties of thin films and multilayers. The BDS values of thin films and multilayers can be as high as MV cm^-1^, which is very helpful for further increasing the recoverable energy density. Thin films of a (0.55−*x*)BiFeO_3_-*x*BaTiO_3_-0.45SrTiO_3_ solid solution (*x* = 0–0.4) containing polymorphic nanodomains showed a recoverable energy density of ~ 110 J cm^−3^ at a BDS ~ 5 MV/cm with an efficiency of ~ 80%, which is the highest among BiFeO_3_-based systems. The achievement of such excellent energy storage properties is very encouraging for applications in low-power, small-size devices. However, for large-scale applications, it is necessary to focus on the fabrication of multilayers, as they deliver high power and are easy to fabricate. Although relatively few studies have been conducted on BiFeO_3_-based multilayers, their energy storage performance is very encouraging (*U*_rec_ ~ 15 J cm^−3^), with a high BDS of ~ 1 MV/cm. More studies should be conducted in the field of BiFeO_3_-based multilayers to optimize structural parameters such as the thickness of dielectric layers and number of layers so that a better energy storage device that can deliver high output power can be fabricated at a low cost.

## References

[1] Goodenough J.B., Abruna H.D., Buchanan M.V. (2007). Basic Research Needs for Electrical Energy Storage. Report of the Basic Energy Sciences Workshop on Electrical Energy Storage, April 2–4, 2007.

[2] Yang L., Kong X., Li F., Hao H., Cheng Z., Liu H., Li J.-F., Zhang S. (2019). Perovskite lead-free dielectrics for energy storage applications. Prog. Mater. Sc..

[3] Kusko A., Dedad J. (2007). Stored energy—Short-term and long-term energy storage methods. IEEE Ind. Appl. Mag..

[4] Yao K., Chen S., Rahimabady M., Mirshekarloo M.S., Yu S., Tay F.E.H., Sritharan T., Lu L. (2011). Nonlinear dielectric thin films for high-power electric storage with energy density comparable with electrochemical supercapacitors. IEEE Trans. Ultrason. Ferroelectr. Freq. Control..

[5] Jow T.R., MacDougall F.W., Ennis J.B., Yang X.H., Schneider M.A., Scozzie C.J., White J.D., MacDonald J.R., Schalnat M.C., Cooper R.A. (2015). Pulsed power capacitor development and outlook. IEEE.

[6] Sarjeant W.J., Zirnheld J., MacDougall F.W. (1998). Capacitors. IEEE Trans. Plasma Sci..

[7] Irvine J.T.S., Sinclair D.C., West A.R. (1990). Electroceramics: Characterization by impedance spectroscopy. Adv. Mater..

[8] Sarjeant W.J., Clelland I.W., Price R.A. (2001). Capacitive components for power electronics. Proc. IEEE..

[9] Bell. A.J. (2008). Ferroelectrics: The role of ceramic science and engineering. J. Eur. Ceram. Soc..

[10] Tan Q., Irwin P., Cao Y. (2006). Advanced dielectrics for capacitors. IEEJ Trans. Fund. Mater..

[11] Chu B.J., Zhou X., Ren K., Neese B., Lin M., Wang Q., Bauer F., Zhang Q.M. (2006). A dielectric polymer with high electric energy density and fast discharge speed. Science.

[12] Khanchaitit. P., Han. K., Gadinski M.R., Li Q., Wang Q. (2013). Ferroelectric polymer networks with high energy density and improved discharged efficiency for dielectric energy storage. Nat. Commun..

[13] Li Q., Han K., Gadinski M.R., Zhang G., Wang Q. (2014). High Energy and Power Density Capacitors from Solution-Processed Ternary Ferroelectric Polymer Nanocomposites. Adv. Mater..

[14] Li Q., Liu F., Yang T., Gadinski M.R., Zhang G., Chen L.-Q., Wang Q. (2016). Sandwich-structured polymer nanocomposites with high energy density and great charge–discharge efficiency at elevated temperatures. Proc. Natl. Acad. Sci. USA.

[15] Peddigari M., Palneedi H., Hwang G.-T., Ryu J. (2019). Linear and nonlinear dielectric capacitors for high-power energy storage capacitor applications. J. Korean Ceram. Soc..

[16] Ravindran P., Vidya R., Kjekshus A., Fjellvåg H., Eriksson O. (2006). Theoretical investigation of magnetoelectric behavior in BiFeO_3_. Phys. Rev. B.

[17] Yang J., Li X., Xiong Z., Wang M., Liu Q. (2020). Environmental Pollution Effect Analysis of Lead Compounds in China Based on Life Cycle. Int. J. Environ. Res. Public Health.

[18] Tchounwou P.B., Yedjou C.G., Patlolla A.K., Sutton D.J. (2012). Heavy metal toxicity and the environment. Molecular, Clinical and Environmental Toxicology.

[19] Zhou H.Y., Liu X.Q., Zhu X.L., Chen X.M. (2017). CaTiO_3_ Linear Dielectric Ceramics with Greatly Enhanced Dielectric Strength and Energy Storage Density. J. Am. Ceram. Soc..

[20] Zhou H.Y., Zhu X.N., Ren G.R., Chen X.M. (2016). Enhanced Energy Storage Density and Its Variation Tendency in CaZr_x_Ti_1−x_O_3_ Ceramics. J. Alloys Compd..

[21] Yang H., Yan F., Lin Y., Wang T. (2017). Enhanced Recoverable Energy Storage Density and High Efficiency of SrTiO_3_-Based Lead-Free Ceramics. Appl. Phys. Lett..

[22] Shay D.P., Podraza N.J., Randall C.A. (2012). High Energy Density, High Temperature Capacitors Utilizing Mn-Doped 0.8CaTiO3–0.2CaHfO3 Ceramics. J. Am. Ceram. Soc..

[23] Lv J., Li Q., Li Y., Tang M., Jin D., Yan Y., Fan B., Jin L., Liu G. (2021). Significantly improved energy storage performance of NBT-BT based ceramics through domain control and preparation optimization. Chem. Eng. J..

[24] Gao F., Dong X., Mao C., Cao F., Wang G. (2011). c/a Ratio-Dependent Energy-Storage Density in (0.9−x)Bi0.5Na0.5TiO3–xBaTiO3–0.1K0.5Na0.5NbO3 Ceramics. J. Am. Ceram. Soc..

[25] Viola G., Ning H., Reece M.J., Wilson R., Correia T.M., Weaver P., Cain M.G., Yan H. (2012). Reversibility in electric field-induced transitions and energy storage properties of bismuth-based perovskite ceramics. J. Phys. D Appl. Phys..

[26] Xu Q., Xie J., He Z., Zhang L., Cao M., Huang X., Lanagan M.T., Hao H., Yao Z., Liu H. (2017). Energy-storage properties of Bi0.5Na0.5TiO3-BaTiO3-KNbO3 ceramics fabricated by wet-chemical method. J. Eur. Ceram. Soc..

[27] Puli V.S., Pradhan D.K., Chrisey D.B., Tomozawa M., Sharma G.L., Scott J.F., Katiyar R.S. (2013). Structure, dielectric, ferroelectric, and energy density properties of (1 − x)BZT–xBCT ceramic capacitors for energy storage applications. J. Mater. Sci..

[28] Puli V.S., Pradhan D.K., Riggs B.C., Chrisey D.B., Katiyar R.S. (2014). Structure, Ferroelectric, Dielectric and Energy Storage Studies of Ba0.70Ca0.30TiO3, Ba(Zr0.20Ti0.80)O3 Ceramic Capacitors. Integr. Ferroelectr..

[29] Zhang Y., Li Y., Zhu H., Fu Z., Zhang Q. (2017). Sintering temperature dependence of dielectric properties and energy-storage properties in (Ba,Zr)TiO3 ceramics. J. Mater. Sci. Mater. Electron..

[30] Zhu C., Wang X., Zhao Q., Cai Z., Chen Z., Li L. (2019). Effects of gtain size and temperature on the energy storage and dielectric tunablity of non-reducible BaTiO_3_-based bulk ceramics. J. Euro. Ceram. Soc..

[31] Liu B., Wang X., Zhao Q., Li L. (2015). Improved energy storage properties of fine crystalline BaTiO_3_ ceramics by coating powders with Al_2_O_3_ and SiO_2_. J. Am. Ceram. Soc..

[32] Qu B., Du H., Yang Z., Liu Q. (2017). Large recoverable energy storage density and low sintering temperature in potassium-sodium niobate-based ceramics for multilayer pulsed power capacitors. J. Am. Ceram. Soc..

[33] Ciuchi I.V., Mitoseriu L., Galassi C. (2016). Antiferroelectric to Ferroelectric Crossover and Energy Storage Properties of (Pb1−xLax)(Zr0.90Ti0.10)1−x/4O3 (0.02 ≤ x ≤ 0.04) Ceramics. J. Am. Ceram. Soc..

[34] Dan Y., Xu H., Zou K., Zhang Q., Lu Y., Chang G., Huang H., He Y. (2018). Energy storage characteristics of (Pb,La)(Zr,Sn,Ti)O_3_ antiferroelectric ceramics with high Sn content. Appl. Phys. Lett..

[35] Jo H.R., Lynch C.S. (2016). A high energy density relaxor antiferroelectric pulsed capacitor dielectric. J. Appl. Phys..

[36] Xu C., Liu Z., Chen X., Yan S., Cao F., Dong X., Wang G. (2016). High charge-discharge performance of Pb0.98La0.02(Zr0.35Sn0.55Ti0.10)0.995O3 antiferroelectric ceramic. J. Appl. Phys..

[37] Li Q., Zhou C., Xu J., Yang L., Zhang X., Zeng W., Yuan C., Chen G., Rao G. (2016). Tailoring antiferroelectricity with high energy-storage properties in Bi0.5Na0.5TiO3–BaTiO3 ceramics by modulating Bi/Na ratio. J. Mater. Sci. Mater. Electron..

[38] Yang H., Yan F., Lin Y., Wang T. (2017). Improvement of dielectric and energy storage properties in SrTiO_3_-based lead-free ceramics. J. Alloys. Compd..

[39] Cao W., Li W., Feng Y., Bai T., Qiao Y., Hou Y., Zhang T., Yu Y., Fei W. (2016). Defect dipole induced large recoverable strain and high energy-storage density in lead-free Na0.5Bi0.5TiO3-based systems. Appl. Phys. Lett..

[40] Zhao L., Liu Q., Gao J., Zhang S., Li J.-F. (2017). Lead-Free Antiferroelectric Silver Niobate Tantalate with High Energy Storage Performance. Adv. Mater..

[41] Zhao L., Gao J., Liu Q., Zhang S., Li J.-F. (2018). Silver Niobate Lead-Free Antiferroelectric Ceramics: Enhancing Energy Storage Density by B-Site Doping. ACS Appl. Mater. Interfaces.

[42] Li J., Jin L., Tian Y., Chen C., Lan Y., Hu Q., Li C., Wei X., Yan H. (2021). Enhanced energy storage performance under low electric field in Sm^3+^ doped AgNbO_3_ ceramics. J. Materiomics.

[43] Zhao Y., Xu J., Zhou C., Yuan C., Li Q., Chen G., Wang H., Yang L. (2016). High energy storage properties and dielectric behavior of (Bi0.5Na0.5)0.94Ba0.06Ti1−x(Al0.5Nb0.5)xO3 lead-free ferroelectric ceramics. Ceram. Int..

[44] Liu Z., Lu T., Ye J., Wang G., Dong X., Withers R., Liu Y. (2018). Antiferroelectrics for energy storage applications: A review. Adv. Mat. Technol..

[45] Palneedi H., Peddigari M., Hwang G.T., Jeong D.W., Ryu J. (2018). High-Performance Dielectric Ceramic Films for Energy Storage Capacitors: Progress and Outlook. Adv. Funct. Mater..

[46] Marqués M.I., Aragó C. (2010). Microscopic model for the formation of nanodomains in relaxor materials. Phys. Rev. B.

[47] Li F., Zhang S., Damjanovic D., Chen L.-Q., Shrout T.R. (2018). Local Structural Heterogeneity and Electromechanical Responses of Ferroelectrics: Learning from Relaxor Ferroelectrics. Adv. Func. Mater..

[48] Kirillov V.V., Isupov V.A. (1973). Relaxation polarization of PbMg_1/3_Nb_2/3_O_3_ (PMN)-A ferroelectric with a diffused phase transition. Ferroelectrics.

[49] Cross L.E. (1987). Relaxor ferroelectrics. Ferroelectrics.

[50] Viehland D., Jang S.J., Cross L.E., Wuttig M. (1990). Freezing of the polarization fluctuations in lead magnesium niobate relaxors. J. Appl. Phys..

[51] Westphal V., Kleemann W., Glinchuk M.D. (1992). Diffuse phase transitions and random-field-induced domain states of the relaxor ferroelectric PbMg_1/3_Nb_2/3_O_3_. Phys. Rev. Lett..

[52] Akbas M.A., Davies P.K. (1997). Domain Growth in Pb(Mg_1/3_Ta_2/3_)O_3_ Perovskite Relaxor Ferroelectric Oxides. J. Am. Ceram. Soc..

[53] Cheng Z.Y., Katiyar R.S., Yao X., Guo A. (1997). Dielectric behavior of lead magnesium niobate relaxors. Phys. Rev. B.

[54] Pirc R., Blinc R. (1999). Spherical random-bond--random-field model of relaxor ferroelectrics. Phys. Rev. B.

[55] Neaton J.B., Ederer C., Waghmare U.V., Spaldin N.A., Rabe K.M. (2005). First-principles study of spontaneous polarization in multiferroic BiFeO_3_. Phys. Rev. B.

[56] Moreau J.M., Michel C., Gerson R., James W.J. (1971). Ferroelectric BiFeO_3_ X-ray and neutron diffraction study. J. Phys. Chem. Solids.

[57] Kubel F., Schmid H. (1990). Structure of a ferroelectric and ferroelastic monodomain crystal of the perovskite BiFeO_3_. Acta Cryst. B.

[58] Fischer P., Polomska M., Sosnowska I., Szymanski M. (1980). Temperature dependence of the crystal and magnetic structures of BiFeO_3_. J. Phys. C Solid State Phys..

[59] Smith R.T., Achenbach G.D., Gerson R., James W.J. (1968). Dielectric Properties of Solid Solutions of BiFeO_3_ with Pb(Ti, Zr)O_3_ at High Temperature and High Frequency. J. Appl. Phys..

[60] Ederer C., Spaldin N.A. (2005). Effect of Epitaxial Strain on the Spontaneous Polarization of Thin Film Ferroelectrics. Phys. Rev. Lett..

[61] Lebeugle D., Colson D., Forget A., Viret M. (2007). Very large spontaneous electric polarization in BiFeO_3_ single crystals at room temperature and its evolution under cycling fields. Appl. Phys. Lett..

[62] Teague J.R., Gerson R., James W.J. (1970). Dielectric hysteresis in single crystal BiFeO_3_. Solid State Commun..

[63] Wang Y.P., Zhou L., Zhang M.F., Chen X.Y., Liu J.-M., Liu Z.G. (2004). Room-temperature saturated ferroelectric polarization in BiFeO_3_ ceramics synthesized by rapid liquid phase sintering. Appl. Phys. Lett..

[64] Ueda K., Tabata H., Kawai T. (1999). Coexistence of ferroelectricity and ferromagnetism in BiFeO_3_–BaTiO_3_ thin films at room temperature. Appl. Phys. Lett..

[65] Palkar V.R., John J., Pinto R. (2002). Observation of saturated polarization and dielectric anomaly in magnetoelectric BiFeO_3_ thin films. Appl. Phys. Lett..

[66] Wang J., Neaton J.B., Zheng H., Nagarajan V., Ogale S.B., Liu B., Viehland D., Vaithyanathan V., Schlom D.G., Waghmare U.V. (2003). Epitaxial BiFeO_3_ Multiferroic Thin Film Heterostructures. Science.

[67] Yun K.Y., Noda M., Okuyama M. (2003). Prominent ferroelectricity of BiFeO_3_ thin films prepared by pulsed-laser deposition. Appl. Phys. Lett..

[68] Lee M.H., Kim D.J., Park J.S., Kim S.W., Song T.K., Kim M.-H., Kim W.-J., Do D., Jeong I.-K. (2015). High-Performance Lead-Free Piezoceramics with High Curie Temperatures. Adv. Mater..

[69] Kumar M.M., Srinivas A., Suryanarayana S.V. (2000). Structure property relations in BiFeO_3_/BaTiO_3_ solid solutions. J. Appl. Phys..

[70] Zhaludkevich D.V., Latushka S.I., Latushka T.V., Sysa A.V., Shaman Y.P., Dronova D.A., Chobot A.N., Chobot G.M., Nekludov K.N., Silibin M.V. (2020). Crystal structure and magnetic properties of (1-x) BiFeO_3_-xBaTiO_3_ ceramics across the phase boundary. Nanomaterials Sci. Eng..

[71] Wei Y., Wang X., Jia J., Wang X. (2012). Multiferroic and piezoelectric properties of 0.65BiFeO3–0.35BaTiO3 ceramic with pseudo-cubic symmetry. Ceram. Int..

[72] Zhou C., Yang H., Zhou Q., Cen Z., Li W., Yuan C., Wang H. (2013). Dielectric, ferroelectric and piezoelectric properties of La-substituted BiFeO_3_–BaTiO_3_ ceramics. Ceram. Int..

[73] Leontsev S.O., Eitel R.E. (2009). Dielectric and Piezoelectric Properties in Mn-Modified (1−x)BiFeO_3_–xBaTiO_3_ Ceramics. J. Am. Ceram. Soc..

[74] Wang T., Jin L., Tian Y., Shu L., Hu Q., Wei X. (2014). Microstructure and ferroelectric properties of Nb_2_O_5_-modified BiFeO_3_-BaTiO_3_ lead-free ceramics for energy storage. Mater. Lett..

[75] Zhu L.-F., Lei X.-W., Zhao L., Hussain M.I., Zhao G.-Z., Zhang B.-P. (2019). Phase structure and energy storage performance for BiFeO_3_–BaTiO_3_ based lead-free ferroelectric ceramics. Ceram. Int..

[76] Wang D., Fan Z., Zhou D., Khesro A., Murakami S., Feteira A., Zhao Q., Tan X., Reaney I.M. (2018). Bismuth ferrite-based lead-free ceramics and multilayers with high recoverable energy density. J. Mater. Chem. A.

[77] Chen Z., Bai X., Wang H., Du J., Bai W., Li L., Wen F., Zheng P., Wu W., Zheng L. (2020). Achieving high-energy storage performance in 0.67Bi1-xSmxFeO3-0.33BaTiO3 lead-free relaxor ferroelectric ceramics. Ceram. Int..

[78] Zheng D., Zuo R., Zhang D., Li Y. (2015). Novel BiFeO_3_–BaTiO_3_–Ba(Mg_1/3_Nb_2/3_)O_3_ Lead-Free Relaxor Ferroelectric Ceramics for Energy-Storage Capacitors. J. Am. Ceram. Soc..

[79] Zheng D., Zuo R. (2017). Enhanced energy storage properties in La(Mg_1/2_Ti_1/2_)O_3_-modified BiFeO_3_-BaTiO_3_ lead-free relaxor ferroelectric ceramics within a wide temperature range. J. Eur. Ceram. Soc..

[80] Liu N., Liang R., Zhou Z., Dong X. (2018). Designing lead-free bismuth ferrite-based ceramics learning from relaxor ferroelectric behavior for simultaneous high energy density and efficiency under low electric field. J. Mater. Chem. C.

[81] Wang D., Fan Z., Li W., Zhou D., Feteira A., Wang G., Murakami S., Sun S., Zhao Q., Tan X. (2018). High Energy Storage Density and Large Strain in Bi(Zn_2/3_Nb_1/3_)O_3_-Doped BiFeO_3_–BaTiO_3_ Ceramics. ACS Appl. Energy Mater..

[82] Wang G., Li J., Zhang X., Fan Z., Yang F., Feteira A., Zhou D., Sinclair D.C., Ma T., Tan X. (2019). Ultrahigh energy storage density lead-free multilayers by controlled electrical homogeneity. Energy Environ. Sci..

[83] Liu N., Liang R., Zhao X., Xu C., Zhou Z., Dong X. (2018). Novel bismuth ferrite-based lead-free ceramics with high energy and power density. J. Am. Ceram. Soc..

[84] Tang M., Yu L., Wang Y., Lv J., Dong J., Guo B., Chen F., Ai Q., Luo Y., Li Q. (2021). Dielectric, ferroelectric, and energy storage properties of Ba(Zn_1/3_Nb_2/3_)O_3_-modfied BiFeO_3_–BaTiO_3_ Pb-Free relaxor ferroelectric ceramics. Ceram. Int..

[85] Sun H., Wang X., Sun Q., Zhang X., Ma Z., Guo M., Sun B., Zhu X., Liu Q., Lou X. (2020). Large energy storage density in BiFeO_3_-BaTiO_3_-AgNbO_3_ lead-free relaxor ceramics. J. Eur. Ceram. Soc..

[86] Yu Z., Zeng J., Zheng L., Rousseau A., Li G., Kassiba A. (2021). Microstructure effects on the energy storage density in BiFeO_3_-based ferroelectric ceramics. Ceram. Int..

[87] Yang H., Qi H., Zuo R. (2019). Enhanced breakdown strength and energy storage density in a new BiFeO_3_-based ternary lead-free relaxor ferroelectric ceramic. J. Eur. Ceram. Soc..

[88] Liu G., Tang M., Hou X., Guo B., Lv J., Dong J., Wang Y., Li Q., Yu K., Yan Y. (2020). Energy storage properties of bismuth ferrite based ternary relaxor ferroelectric ceramics through a viscous polymer process. Chem. Eng. J..

[89] Li Q., Ji S., Wang D., Zhu J., Li L., Wang W., Zeng M., Hou Z., Gao X., Lu X. (2021). Simultaneously enhanced energy storage density and efficiency in novel BiFeO_3_-based lead-free ceramic capacitors. J. Eur. Ceram. Soc..

[90] Ji S., Li Q., Wang D., Zhu J., Zeng M., Hou Z., Fan Z., Gao X., Lu X., Li Q. (2021). Enhanced energy storage performance and thermal stability in relaxor ferroelectric (1-x)BiFeO3-x(0.85BaTiO3-0.15Bi(Sn0.5Zn0.5)O3) ceramics. J. Am. Ceram. Soc..

[91] Lu Z., Wang G., Bao W., Li J., Li L., Mostaed A., Yang H., Ji H., Li D., Feteira A. (2020). Superior energy density through tailored dopant strategies in multilayer ceramic capacitors. Energy Environ. Sci..

[92] Qi H., Xie A., Tian A., Zuo R. (2020). Superior Energy-Storage Capacitors with Simultaneously Giant Energy Density and Efficiency Using Nanodomain Engineered BiFeO_3_-BaTiO_3_-NaNbO_3_ Lead-Free Bulk Ferroelectrics. Adv. Energy Mater..

[93] Correia T.M., McMillen M., Rokosz M.K., Weaver P.M., Gregg J.M., Viola G., Cain M.G. (2013). A Lead-Free and High-Energy Density Ceramic for Energy Storage Applications. J. Am. Ceram. Soc..

[94] Pan H., Zeng Y., Shen Y., Lin Y.-H., Ma J., Li L., Nan C.-W. (2017). BiFeO_3_–SrTiO_3_ thin film as a new lead-free relaxor-ferroelectric capacitor with ultrahigh energy storage performance. J. Mater. Chem. A.

[95] Hu Z., Ma B., Koritala R.E., Balachandran U. (2014). Temperature dependent energy storage properties of antiferroelectric Pb_0_. 96La0.04Zr0.98Ti0.02O3 thin films. Appl. Phys. Lett..

[96] Pan H., Ma J., Ma J., Zhang Q., Liu X., Guan B., Gu L., Zhang X., Zhang Y.-J., Li L. (2018). Giant energy density and high efficiency achieved in bismuth ferrite-based film capacitors via domain engineering. Nat. Commun..

[97] Pan H., Li F., Liu Y., Zhang Q., Wang M., Lan S., Zheng Y., Ma J., Gu L., Shen Y. (2019). Ultrahigh–energy density lead-free dielectric films via polymorphic nanodomain design. Science.

[98] Kursumovic A., Li W.-W., Cho S., Curran P.J., Tjhe D.H.L., MacManus-Driscoll J.L. (2020). Lead-free relaxor thin films with huge energy density and low loss for high temperature applications. Nano Energy.

[99] Yan F., Shi Y., Zhou X., Zhu K., Shen B., Zhai J. (2020). Optimization of polarization and electric field of bismuth ferrite-based ceramics for capacitor applications. Chem. Eng. J..

[100] Wang G., Lu Z., Li J., Ji H., Yang H., Li L., Sun S., Feteira A., Yang H., Zuo R. (2020). Lead-free (Ba,Sr)TiO_3_—BiFeO_3_ based multilayer ceramic capacitors with high energy density. J. Eur. Ceram. Soc..

[101] Wang G., Lu Z., Yang H., Ji H., Mostaed A., Li L., Wei Y., Feteira A., Sun S., Sinclair D.C. (2020). Fatigue resistant lead-free multilayer ceramic capacitors with ultrahigh energy density. J. Mater. Chem. A.

[102] Qi X., Dho J., Tomov R., Blamire M.G., MacManus-Driscoll J.L. (2005). Greatly reduced leakage current and conduction current mechanism in aliovalent-ion-doped BiFeO_3_. Appl. Phys. Lett..

[103] Hu G.D., Fan S.H., Yang C.H., Wu W.B. (2008). Low leakage and current and enhanced ferroelectric properties of Ti and Zn codoped BiFeO_3_ thin films. Appl. Phys. Lett..

[104] Singh S.K., Maruyama K., Ishiwara H. (2007). Reduced leakage current in La and Na codoped BiFeO_3_ thin films. Appl. Phys. Lett..

[105] Hu G.D., Cheng X., Wu W.B., Yang C.H. (2007). Effect of Gd substitution on structure and ferroelectric properties of BiFeO3 thin films prepared using metal organic decomposition. Appl. Phys. Lett..

[106] Veerapandian V., Benes F., Gindel T., Deluca M. (2020). Strategies to develop energy storage properties of perovskite lead free relaxor ferroelectrics: A review. Materials.

[107] Ray S. (2013). An Introduction to High Voltage Engineering.

[108] Chen I.W., Wang X.H. (2000). Sintering dense nanocrystalline ceramics without final-stage grain-growth. Nature.

[109] Kim C., Pilania G., Ramprasad R. (2016). From organized high-throughput data to phenomenological theory using machine learning: The example of dielectric breakdown. Chem. Mater..

[110] Moubah R., Schmerber G., Rousseau O., Colson D., Viret M. (2012). Photoluminescence Investigation of Defects and Optical Band Gap in Multiferroic BiFeO_3_ Single Crystals. Appl. Phys. Express.

[111] Walker J., Simons H., Alikin D.O., Turygin A.P., Shur V.Y., Kholkin A.L., Ursic H., Bencan A., Malic B., Nagarajan V. (2016). Dual strain mechanisms in a leadfree morphotropic phase boundary ferroelectric. Sci. Rep..

